# Unraveling Molecular and Functional Responses Across 3 Lung Injury Models to Expand the Donor Lung Pool

**DOI:** 10.1097/TP.0000000000005353

**Published:** 2025-02-19

**Authors:** Gabriel Hirdman, Martin Stenlo, Nicholas Burdon Bèchet, Anna Niroomand, Margareta Mittendorfer, Qi Wang, Dag Edström, Haider Ghaidan, Sven Kjellström, Leif Pierre, Franziska Olm, Snejana Hyllén, Sandra Lindstedt

**Affiliations:** 1 Department of Cardiothoracic Anaesthesia and Intensive Care and Cardiothoracic Surgery and Transplantation, Skåne University Hospital, Lund University, Lund, Sweden.; 2 Wallenberg Center for Molecular Medicine, Lund University, Lund, Sweden.; 3 Lund Stem Cell Center, Lund University, Lund, Sweden.; 4 Department of Clinical Sciences, Lund University, Lund, Sweden.; 5 Rutgers Robert University, New Brunswick, NJ.; 6 BioMS-Swedish National Infrastructure for Biological Mass Spectrometry, Lund University, Lund, Sweden.

## Abstract

**Background.:**

Lung transplantation remains hampered by a scarcity of viable donor lungs, partially attributed to donor lung injuries.

**Methods.:**

Three porcine lung injury models were studied: infection-induced using lipopolysaccharide (n = 7), aspiration-induced using endotracheal gastric content (n = 7), and injury using lavage and harmful ventilation (ventilator-induced lung injury; n = 7). Molecular and functional changes from before and after the establishment of lung injury were examined with histopathology, immunohistochemistry, cytokine levels, hemodynamics, and mass spectrometric analysis of lung tissue. The respiratory tract lining fluid was analyzed using exhaled breath particles.

**Results.:**

T-cell proliferation and suppression of complement activation were unique to the gastric injury, whereas the ventilator-induced lung injury group displayed a unique activation of monocyte chemotaxis. The lipopolysaccharide injury exhibited an activation of stress response proteins. Alterations in the extracellular matrix, particularly the degradation of collagen type IV and increased elastin expression, were identified as a consistent indicator of acute lung injury. Additionally, increases in exhaled particles and differential expression of proteins in the respiratory tract lining fluid correlated with deteriorating lung function.

**Conclusions.:**

Molecular analysis of the lung indicated distinct key differences and similarities of donor lung injury phenotypes. Analysis of various donor lung injuries suggests a heightened emphasis on the extracellular matrix for the restoration and rejuvenation of damaged donor lungs.

## Graphical abstract

**Figure:**
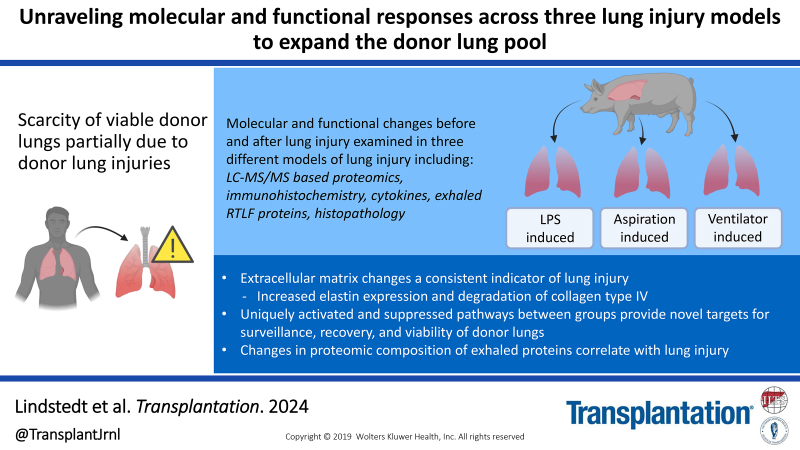

## INTRODUCTION

Lung transplantation (LTx) is the only treatment option for patients with end-stage lung disease. Despite its life-saving potential, the procedure faces significant challenges, including a limited availability of viable donor organs. Up to 80% of potential donor lungs are discarded because of concerns about donor lung injury and subsequent risk of primary graft dysfunction (PGD), in stark contrast to the 17% discard rate observed in kidneys.^1-3^ Common reasons to discard donor lungs include the circumstances leading to the death or injury to the lungs during the time on mechanical ventilation, including neurogenic edema, gastric aspiration, infection, ventilator-induced lung injury (VILI), and trauma.^4^ There is currently no treatment for the regeneration of these discarded donor lungs; hence, any means to restore lung function and ameliorate injury would have a significant impact on donor lung shortages.^5^ To develop targeted and effective treatments to restore discarded donor lungs effectively, a deeper understanding of various injury causes is needed.

Sampling directly from the lung poses significant challenges, often involving invasive procedures such as biopsies and bronchoalveolar lavage fluid (BALF). Although lung biopsies offer unique insights into donor lung quality, transbronchial lung biopsy sampling areas are very small, and BALF contains contaminants from the entire respiratory tract. A noninvasive alternative to tissue sampling involves the analysis of exhaled breath particles (EBPs), which originate from the respiratory tract lining fluid covering the epithelial surface of the entire distal regions of the lung.^6,7^ The rate of EBP production notably increases in response to lung injury, which is hypothesized to be due to heightened capillary leakage and the accumulation of fluids within the alveoli.^8-10^ However, the analysis of transbronchial lung biopsies and EBP collection does encounter limitations due to the limited physical material collected during sample acquisition.

Significant advances in global protein profiling through the use of nontargeted quantitative proteomics with liquid chromatography-tandem mass spectrometry have emerged as a powerful tool in disease characterization.^11^ Particularly, data-independent acquisition (DIA), with the capacity of accurate and reproducible untargeted quantification of complex protein mixtures, has significant potential for the identification of fundamental mechanisms and novel biomarkers of diseases.^12^ These technologies can be leveraged to detect proteomic biomarkers and biological pathways from previously challenging sampling sources, especially in small samples such as EBP and lung biopsies. The only shortcoming is that this technique explores global tissue protein changes and is unable to resolve spatial-localized changes that may only arise in specific structures and/or regions. Thus, to supplement the global protein profiling, we have established an advanced imaging approach to quantitatively assess protein changes in focused structures such as the airways and blood vessels.

In the present study, we studied 3 distinct porcine models of lung injury, representing the commonly encountered clinical scenarios of infection, aspiration, and VILI. We used a comprehensive proteomic approach, using mass spectrometry and immunofluorescent staining on both EBP and lung biopsy samples. Our objective was to characterize the proteomic signature associated with each of these acute lung injury (ALI) models.

## MATERIALS AND METHODS

Methods, described in greater detail, and bioinformatics sections are available in **Supplemental Materials and Methods** (**SDC,** http://links.lww.com/TP/D231).

### Animal Preparation

Twenty-one male and female Yorkshire pigs, averaging 33.5 ± 1.4 kg, were stratified into 3 groups and premedicated. Anesthesia was maintained using intravenous ketamine, fentanyl, and midazolam. Due to the increased length of the airway in pigs, a secure airway was established with a tracheostomy, and invasive monitoring was achieved via right carotid and pulmonary artery catheters. Hemodynamic stability was maintained with norepinephrine, dobutamine, and fluids. Detailed medication protocols are available in the **Supplemental Materials and Methods** (**SDC,** http://links.lww.com/TP/D231). The study was approved by the local Ethics Committee for Animal Research (DNR 5.2.18-4903/16 and DNR 5.2.18-8927/16). All animals received care according to the US Principles of Laboratory Animal Care of the National Society for Medical Research, Guide for the Care and Use of Laboratory Animals, National Academies Press (1996).

### Induction of ALI in the 3 Models

ALI was induced using 3 different models. In the lipopolysaccharide (LPS) group (n = 7), LPS was administered intravenously at 0.5 µg/kg/min for 1 h. In the gastric aspiration group (n = 7), gastric content standardized to a pH of 2 was bronchoscopically instilled at 4 mL/kg equally distributed between different lung lobes. The VILI group (n = 7) underwent a double-hit model: 30 mL/kg saline lavage followed by 120 min of harmful mechanical ventilation.

### Biopsies, Histology, Cytokines, Hemodynamics, EBPs, and Immunofluorescence Staining

Lung biopsies from the right lower lobe were obtained at baseline and at the study endpoint and snap frozen. For histological analysis, biopsies were fixed in 10% formalin and paraffin-embedded. Plasma samples were taken before lung injury (BLI), upon lung injury, and at the endpoint, whereas BALF was taken BLI and at the endpoint. Hemodynamic data and arterial blood gases were assessed every 30 min. A PExA 2.0 device (PExA, Gothenburg, Sweden), connected to the expiratory limb of the mechanical ventilator, was used for EBPs collection at BLI and endpoint while particle concentrations were continuously monitored. The PExA device collects particles onto a 0.45-µm polytetraflouroethylene filter (Merck Millipore Ltd, Cork, Ireland) using an impactor while particle concentrations are measured using an optical particle counter and airflow monitor, described in detail elsewhere.^13,14^ Histological scoring was done by 3 blinded scorers using hematoxylin and eosin-stained slides (Histolab, Sweden). Immunostaining was accomplished with primary antibodies against elastin and smooth muscle actin, followed by fluorescently labeled secondary antibodies. Samples were costained with 4′,6-diamidino-2-phenylindole, and tomato lectin and subsequently imaged using confocal microscopy. Alveolar morphology was analyzed as previously described.^15^ Statistical analysis was performed in GraphPad Prism version 10.0.2 (Prism, USA).

### Mass Spectrometry Acquisition and Bioinformatics

Homogenized lung tissue and EBP membranes were solubilized and digested using an S-TRAP-based digestion protocol. Mass spectra were acquired using a PaSEF 4D DIA method. Raw DIA data were processed using DIA-NN version 1.8.1. Differential gene expression, hierarchical clustering, gene set enrichment analysis, and extracellular matrix (ECM) annotation were performed in R version 4.3.1 using various packages (MS-DAP, pheatmap, clusterprofiler, and MatrisomeAnalyseR).^16-19^ Differentially expressed proteins (DEPs) were defined as false discovery rate (FDR)-adjusted *P* values (q-value) of <0.05 and fold change thresholds estimated by bootstrap analysis.

## RESULTS

### Hemodynamics, Blood Gases, and Ventilation Parameters in the 3 Lung Injury Models

Throughout the experiments, blood gases, hemodynamic parameters, and ventilatory parameters were recorded, as shown in **Tables S1** (**SDC,** http://links.lww.com/TP/D233), **S2** (**SDC,** http://links.lww.com/TP/D234), and **S3** (**SDC,** http://links.lww.com/TP/D235). All 3 injury groups exhibited a decline in blood pH and pO_2_ and a simultaneous increase in pCO_2_ indicative of ALI. Acute respiratory distress syndrome was achieved after an average of 90 ± 42.4 min in the LPS group, 30 ± 75.1 min in the VILI group, and after 30 ± 15.0 min in the gastric group. Endpoint, or time of death, was reached after 115.7 ± 42.4 min in the LPS group, 377.1 ± 38.8 min in the VILI group, and 300.0 ± 58.1 min in the gastric group. Notably, the VILI group showed more pronounced impairment in gas exchange parameters at the endpoint, exemplified by significantly higher end pCO_2_ (*P* < 0.0001). Furthermore, lung function deteriorated across the 3 models, characterized by reduced dynamic compliance and increased peak inspiratory pressure, again with significantly worse values for the VILI group. The PaO_2_/FiO_2_ ratio decreased rapidly in the VILI and gastric group, whereas the LPS group exhibited a more gradual decline.

Along with declining pulmonary function, hemodynamic changes occurred in cardiac output, mean pulmonary pressure, and pulmonary vascular resistance. The LPS group experienced the most severe systemic changes, with a significantly higher cardiac output compared with the VILI (*P* = 0.0040) and gastric (*P* = 0.0005) groups.

### Histological Characteristics Confirming ALI in all 3 Lung Injury Models

**FIGURE 1.**
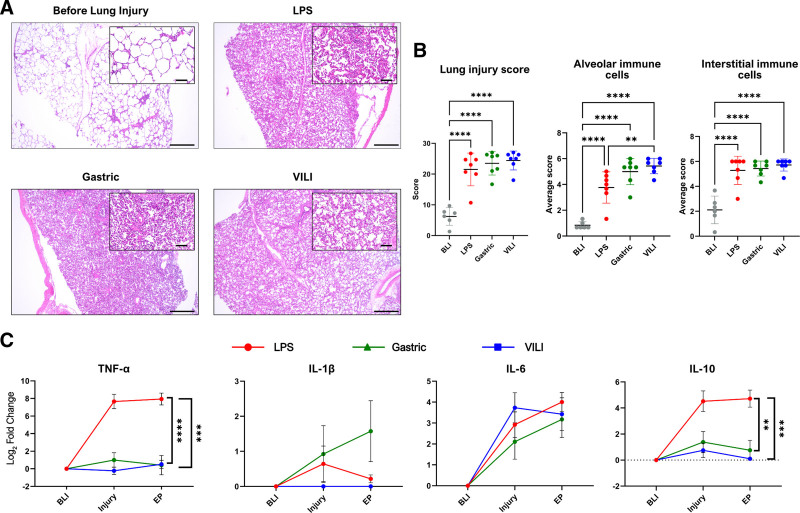
Confirmation of acute lung injury across the 3 lung injury models. A, Representative images of lung biopsies with H&E staining taken BLI and at the endpoint of the experiment. The BLI sample demonstrates normal lung histology exemplified by open airspaces. The scale bar in the larger image represents 500 µm, and the callout shows a magnified view of the tissue with the scale bar representing 100 µm. B, Results of blinded scoring of histological samples from BLI and lung injury samples across the 3 groups. Results are shown as summed lung injury score, average score (from 1 to 6) for the number of neutrophils/immune cells in the alveolar airspace, and the number of neutrophils/immune cells in the interstitial space. C, Plasma cytokine changes over the experimental timeline expressed as log_2_ fold change from BLI for TNF-α, IL-1ß, IL-6, and IL-10. D–G, Representative images of the bronchoalveolar scaffold (LEA: white, DAPI: blue) from BLI and lung injury samples. H, Quantification of bronchoalveolar scaffold density BLI and in established lung injury. I, Representative images of individual alveoli from BLI and lung injury samples. J–L, Quantification of alveolar morphology metrics at BLI and in acute lung injury. Statistically significant differences were tested with a 1-way ANOVA with Tukey multiple comparisons for the lung injury scores, and for the plasma cytokine changes, a 2-way ANOVA with Tukey multiple comparisons was used (only endpoint statistically significant differences are shown). Results are shown as mean ± SEM. *P < 0.05, **P < 0.01, ***P < 0.001, ****P < 0.0001. BLI, before lung injury; DAPI, 4′,6-diamidino-2-phenylindole; H&E, hematoxylin and eosin; IL, interleukin; LEA, *Lycopersicon esculentum* lectin; TNF-α, tumor necrosis factor-α.

All groups exhibited significant increases in lung injury scores compared with BLI, with no differences observed between groups. Representative images of the lung before injury show normal tissue without anomalies, whereas endpoint samples demonstrated signs of lung injury, including alveolar damage and thickening of the alveolar-capillary barriers (Figure 1A). Interestingly, the LPS group showed a lower number of immune cells in the alveoli at the endpoint compared with the other groups, with no such differences seen in the interstitium (Figure 1B).

### Cytokine Changes Measured in Plasma and BALF Differed Between the Injury Models

The LPS group exhibited substantial increases in cytokine levels, particularly tumor necrosis factor alpha (TNF-α) and interleukin (IL)-10, as compared with the VILI and gastric groups (*P* < 0.0001 and *P* = 0.0004, respectively, for TNF-α; Figure 1C). IL-1β exhibited minor increases in the LPS and gastric group but remained under the limit of detection in the VILI group. IL-6 concentration levels increased significantly from BLI across all groups, with no significant differences at the endpoint between the groups.

BALF cytokine levels generally mirrored those of plasma, with a few exceptions. TNF-α levels exhibited a log_2_ fold change increase from BLI of 2.39 ± 4.23 and 1.91 ± 2.55 for the LPS and gastric group, respectively, at the endpoint. In contrast, the VILI group exhibited a log_2_ fold change decrease of –1.26 ± 2.82, leading to a significant difference between the LPS and VILI groups (*P* = 0.0152). The large increases in IL-10 plasma levels in the LPS group were furthermore not seen in BALF, where the LPS group exhibited the lowest increase with a log_2_ fold change increase of 1.50 ± 1.90, whereas the VILI and gastric groups exhibited an increase of 4.89 ± 4.42 and 12.28 ± 0.00, respectively.

### Proteomic Analysis of Tissue Revealed Distinct Upregulation of Immune System–related Proteins and Changes to the ECM Composition After Lung Injury

**FIGURE 2.**
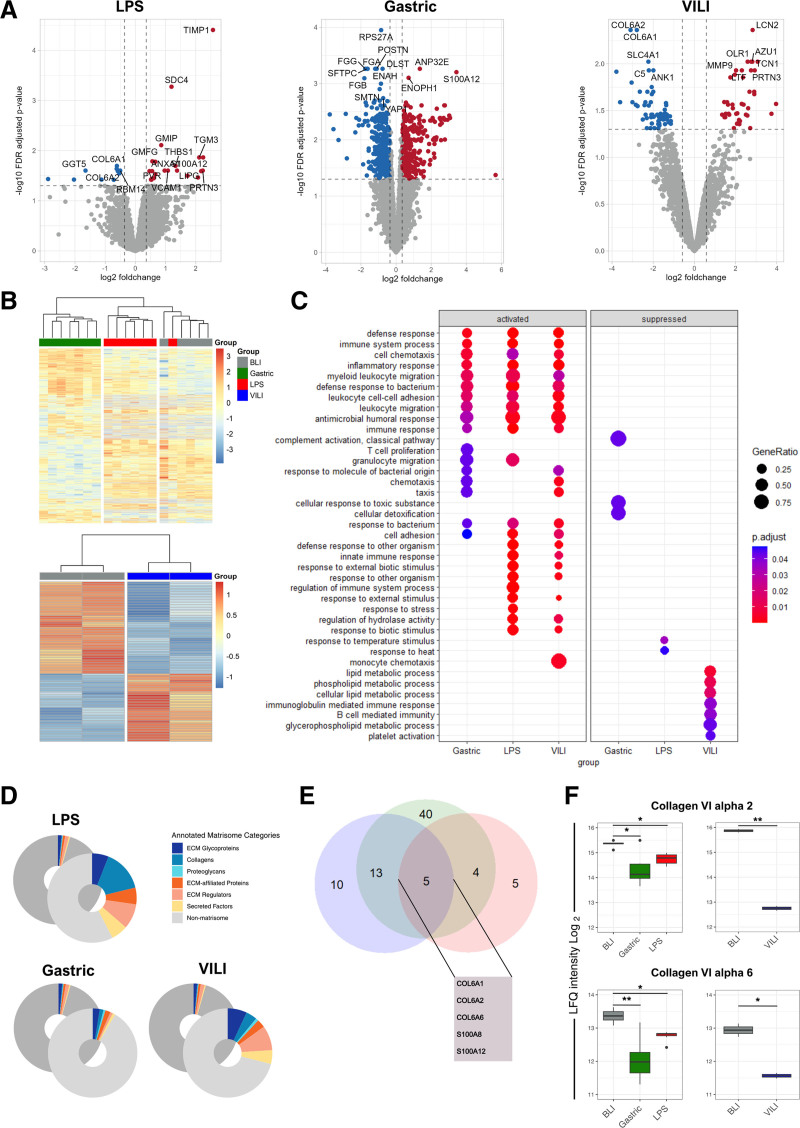
Proteomic expression analysis of lung injury tissue. A, Volcano plots of differentially expressed proteins between BLI and at the endpoint for the LPS group, gastric group, and VILI group. Significantly overexpressed proteins at the endpoint are highlighted in red, significantly downregulated proteins are highlighted in blue, and nonsignificant proteins in gray, with the top 12 most significant proteins labeled. B, Heatmaps of differentially expressed proteins with unsupervised hierarchical clustering showing a clear separation between groups at the endpoint and BLI. C, Gene set enrichment analysis showing significantly enriched pathways compared at the endpoint in the 3 groups. D, Circular diagrams showing the proportion of proteins identified as components of the ECM among all proteins observed at the endpoint (represented in the rear diagram) and among all proteins that exhibited significant differential expression (shown in the foreground diagram) across the 3 study groups. ECM-related proteins constituted a disproportionately large fraction of differentially expressed proteins relative to the total number of proteins identified. E, Venn diagram depicting the overlap of significantly differentially expressed ECM-related proteins between the 3 groups with the callout annotating the 5 proteins that were found to be significant in all 3 groups. These were COL6A1, COL6A2, COL6A6, S100A8, and S100A12. F, COL6A2 and COL6A6 intensity in LC-MS/MS analysis at BLI and the endpoint for the 3 groups. Data analysis was performed in R using DEqMS for differential expression analysis. Significantly differentially expressed proteins were defined as FDR-corrected *P* value of < 0.05 and boot-strapped inferred log2 fold change cutoffs. Results are shown as mean ± SD. *P < 0.05, **P < 0.01, ***P < 0.001, ****P < 0.0001. BLI, before lung injury; COL6A, collagen type VI alpha; ECM, extracellular matrix; FDR, false discovery rate; LC-MS/MS, liquid chromatography-tandem mass spectrometry; LPS, lipopolysaccharide; S100A8, calgranulin A; S100A12, calgranulin C; VILI, ventilator-induced lung injury.

Liquid chromatography-tandem mass spectrometry (LC-MS/MS) analysis of tissue samples identified a mean of 6985 ± 442 proteins per sample. Comparisons between BLI and endpoint samples revealed 736 significantly DEPs in the gastric cohort and 100 and 33 DEPs in the VILI and LPS cohorts, respectively (Figure 2A; **Table S4**, **SDC**, http://links.lww.com/TP/D236). The hierarchical clustering of all DEPs clearly differentiated samples by injury, indicating distinct proteomic profiles compared with BLI in different ALI models (Figure 2B). Activated and suppressed pathways involved inflammatory responses, leukocyte adhesion, and migration. Specifically, the gastric group showed heightened T-cell proliferation and reduced complement activation, whereas the LPS group exhibited an increased innate immune response. The VILI group presented a mix of these profiles, with additional suppression in lipid metabolism and B-cell immunity (Figure 2C; **Table S5, SDC,** http://links.lww.com/TP/D237). A disproportionate number of DEPs belonged to the ECM. This is best exemplified in the LPS group, where ECM-related proteins accounted for 42.4% of all DEPs as opposed to just 4.5% of all identified proteins (Figure 2D). Changes in the ECM were characterized by the loss of ECM glycoproteins, collagen, and ECM-affiliated proteins and the upregulation of ECM regulators (**Figure S1A–C, SDC,** http://links.lww.com/TP/D231). Five DEP ECM-related proteins were found in all 3 ALI models: collagen type VI chains (COL6A1, A2, and A6) and proteins S100A8 and S100A12 (Figure 2E and F).

### Immunofluorescence Imaging Reveals Injury-specific Pulmonary Structural Remodeling and Generalized Pneumocyte Damage

**FIGURE 3.**
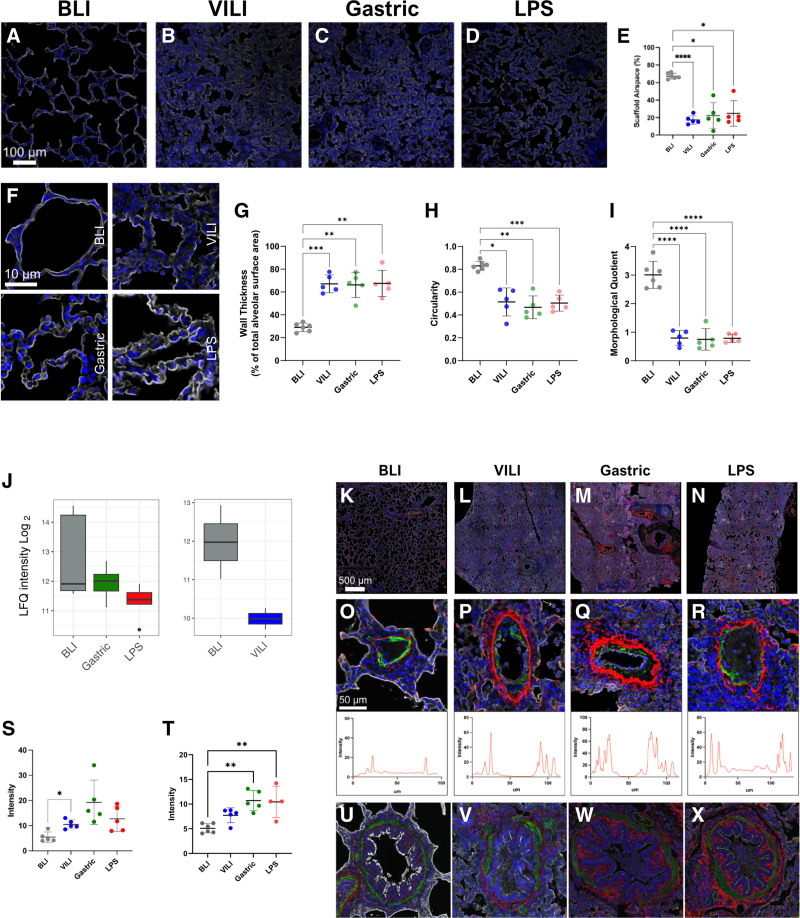
Gastric and LPS-induced injuries upregulate elastin expression across lung structures. A–D, Large field of view representative images of lung biopsies from BLI and lung injury samples stained for SMA (green) and elastin (red). E–H, Representative images of the individual blood vessels in cross-section from BLI and lung injury samples stained for SMA and elastin. Intensity plots of elastin expression across the vessel wall are shown beneath each image. I, Quantification of elastin intensity surrounding blood vessels from BLI and lung injury samples. J–M, Representative images of the individual intrapulmonary bronchioles in cross-section from BLI and lung injury samples stained for SMA and elastin. N, Quantification of elastin intensity surrounding intrapulmonary bronchioles from BLI and lung injury samples. One-way ANOVA with Tukey multiple corrections. Results are shown as mean ± SD. *P < 0.05, **P < 0.01, ***P < 0.001, ****P < 0.0001. BLI, before lung injury; DAPI, 4′,6-diamidino-2-phenylindole; LPS, lipopolysaccharide; SMA, smooth muscle actin; VILI, ventilator-induced lung injury.

To elucidate the microscale spatial biology in various lung injury models, we performed multiplex immunofluorescence imaging on pre- and postinjury lung samples. All injury types showed disrupted tissue morphology, marked by increased bronchoalveolar scaffold coverage and reduced airspace, with a uniform increase in alveolar wall thickness (Figure 3A–I). Despite these similarities, our investigation into the ECM component elastin revealed injury-specific patterns of structural remodeling not found in global proteomics expression measurements (Figure 3J–X). Elastin was particularly enriched in gastric and LPS-induced injuries, as evidenced by intensified fluorescence and vessel wall thickening (Figure 3S). Bronchioles in injured lungs also displayed increased elastin, most notably in the LPS and gastric groups (Figure 3T).

**FIGURE 4.**
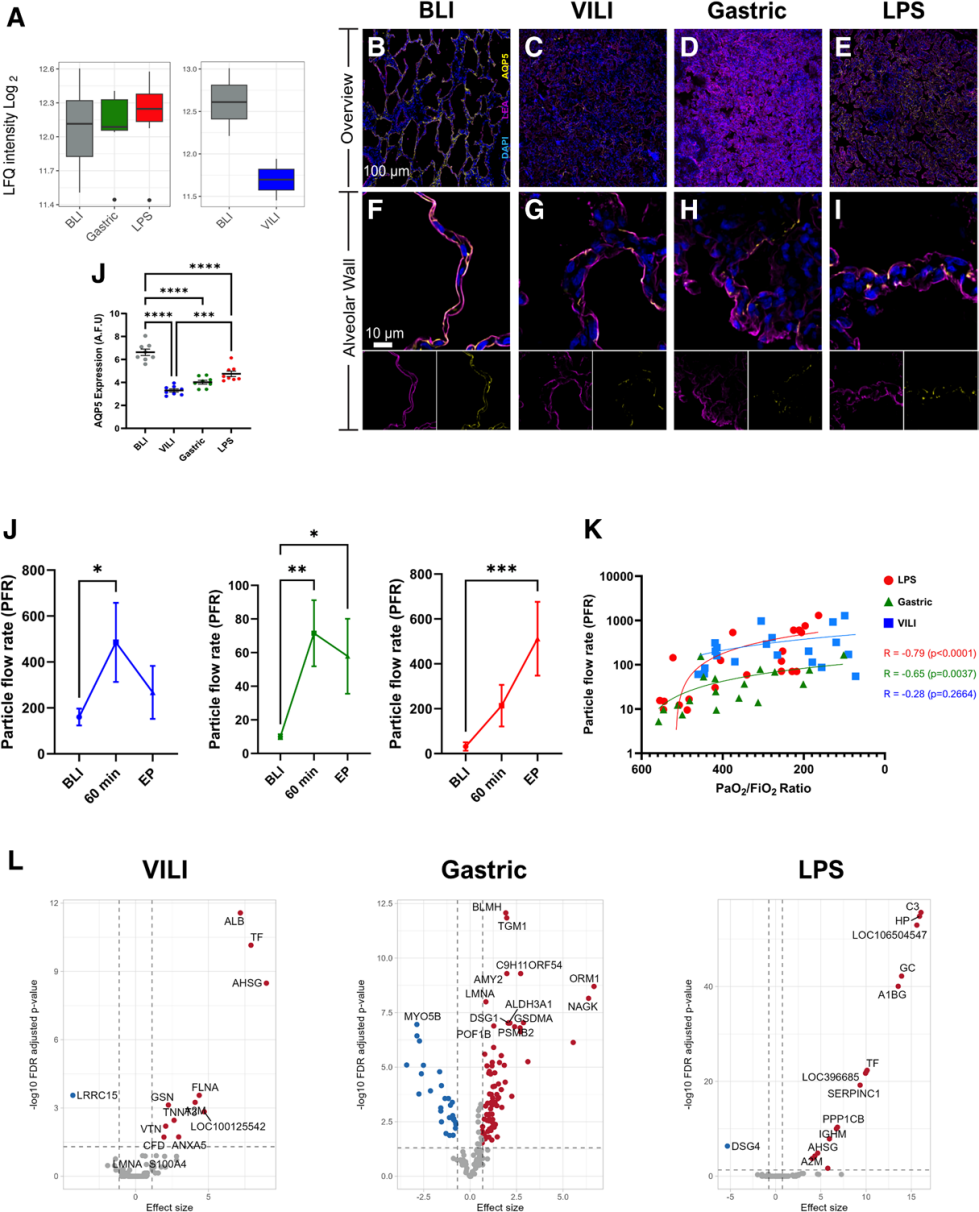
Acute lung injury reduces the expression of AQP5 in pneumocytes and significantly increases PFR. A–D, Representative images of the bronchoalveolar scaffold from BLI and lung injury samples stained for AQP5. E–H, Representative images of individual alveolar walls from BLI and lung injury samples stained for AQP5. I, Quantification of AQP5 intensity in the alveoli from BLI and lung injury samples. J, PFR from the airways during the experimental timeline in animals who received LPS, gastric aspiration, and harmful ventilation (VILI). K, Correlation between PaO_2_/FiO_2_ ratios and PFR BLI, 60 min, and endpoint. A simple linear regression line is plotted for illustrative purposes, and R values are shown. L, Volcano plots of differentially expressed proteins from BLI in the LPS, gastric, and VILI groups. Significantly overexpressed proteins in lung injury are highlighted in red, significantly downregulated proteins are highlighted in blue, and nonsignificant proteins in gray. One-way ANOVA with Tukey multiple corrections was used for AQP5 expression differences. Friedman test with Dunn multiple comparisons was used for the individual PFR timelines, and Spearman *r* correlation was used for PaO_2_/FiO_2_ with PFR. Mass spectrometry data were performed in R using MSqRob2 for differential expression analysis. Significantly differentially expressed proteins were defined as FDR corrected *P* value of <0.05 and boot-strapped inferred log2 fold change cutoffs. Results are shown as mean ± SD. *P < 0.05, **P < 0.01, ***P < 0.001, ****P < 0.0001. AQP5, aquaporin-5; BLI, before lung injury; DAPI, 4′,6-diamidino-2-phenylindole; FDR, false discovery rate; LEA, *Lycopersicon esculentum* lectin; LPS, lipopolysaccharide; PFR, particle flow rate; VILI, ventilator-induced lung injury.

Regarding aquaporin-5 (AQP5) expression, a known mediator of fluid homeostasis, we observed no significant differences in expression in the global proteomics data between healthy and injured lungs in the different groups (Figure 4A). However, imaging data showed a continuous distribution in healthy lungs, whereas injured lungs showed patchy AQP5 presence, most severely affected in VILI cases (Figure 4B–J). Collectively, these findings suggest that although lung injuries share morphological alterations, they diverge in molecular and structural remodeling, indicating distinct pathophysiological mechanisms.

### Particle Flow Rate and Proteomic Composition of EBPs in the 3 Lung Injury Models

Particle flow rate (PFR) increased significantly during the experimental timeline in all 3 ALI models. Significant differences were seen already at 60 min postinduction for the VILI and gastric group and at the endpoint for the LPS group (Figure 4J). To investigate whether increases in PFR correlated with the onset of ALI, PFR was compared with PaO_2_/FiO_2_ ratios. This revealed a significant correlation in the LPS group (*r* = –0.79, *P* < 0.0001) and in the gastric group (*r* = –0.65, *P* = 0.0037) but not in the VILI group (*r* = 0.26, *P* = 0.2664; Figure 4K).

LC-MS/MS analysis of EBPs identified an average of 576 ± 135 proteins. Differential expression analysis between BLI and endpoint samples revealed 17 DEPs in the LPS group, whereas the VILI and gastric cohort exhibited 12 and 106 DEPs, respectively (Figure 4L; **Table S6, SDC,** http://links.lww.com/TP/D238). Protein-protein interaction analysis revealed strong interactions and associations among DEPs in each group as well as significantly enriched pathways (**Figure S2A–C, SDC,** http://links.lww.com/TP/D231). These included neutrophil and platelet degranulation pathways as well as the complement and coagulation cascade in the LPS and VILI groups (FDR, 1.42 × 10^–7^ and 6.53 × 10^–6^, respectively). In the gastric cohort, 30 DEPs, annotated as belonging to the cell junction (FDR, 8.08 × 10^–7^), were found upregulated in EBP, possibly evidence of increased excretion of cell junction-related proteins. Notably, a majority of DEPs in gastric EBP belonged to the cytoplasm as well as the alimentary canal. This could be the result of gastric aspiration productions remaining along with subsequent rupturing of the cellular membrane and release of cytoplasmic constituents.

## DISCUSSION

The field of LTx is currently hindered by a critical shortage of donor organs, exacerbated by a notably low utilization rate of available lungs. This challenge is compounded by the varied nature of lung injuries that afflict donor grafts, complicating the development of effective treatments for organ recovery. To advance our understanding of targeted therapies for specific injury types, we used a porcine model to replicate lung injury through 3 distinct mechanisms. This approach enabled us to examine proteomic and molecular changes in the lung parenchyma and the respiratory tract lining fluid, thereby mirroring the most common clinical conditions that render donor lungs unsuitable for transplantation. These conditions include infection, aspiration, and VILI. By using untargeted LC-MS/MS analysis, we were able to analyze changes in donor lung injury encompassing thousands of proteins and not limited to a predefined set of proteins, enabling unbiased discovery of changes in early lung injury. In this study, we were thus able to successfully identify distinct alterations in structure, pathways, and biomarkers associated with each injury type.

The study of different ALI phenotypes remains important given the current gaps in our knowledge of specific pathophysiology. ALI is a known consequence of the onset of edema, attributable to the disruption of alveolar and epithelial barrier integrity, which permits protein-rich fluid buildup in the alveolar space.^20^ Various mechanisms have previously been implicated in the degradation of the alveolar epithelium, including cellular apoptosis, disruption of tight junctions, and alterations in ECM components.^21^ In this study, proteins related to the ECM were disproportionately represented among the DEPs. The ECM plays a pivotal role in maintaining barrier functions and regulating the trafficking of immune cells, fluids, and molecules.^22^ Changes in the ECM often precede inflammatory reactions.^23^ Our data suggest that similar ECM alterations could serve as an early indicator of injury and ensuing risk of PGD. In all 3 of our models, multiple COL6 alpha chains were significantly underexpressed. A highly expressed protein in the lung basement membrane, COL6 plays an important role in tissue maintenance and cell adhesion, migration, and apoptosis.^24^ COL6 can be proteolytically degraded by matrix metalloproteinase-2and matrix metalloproteinase-9, which were, in turn, also found upregulated in our study in both the gastric and VILI groups. Proteolytic fragments of COL6 have been proposed as a disease marker for fibrosis.^25^ However, our data show that it could be used even earlier to detect early stages of ALI. Future studies should be conducted to evaluate COL6 fragments in donor lungs as a prognostic marker of future graft health.

To explore injury-specific ECM alterations in a more spatially resolved manner, we also carried out high-resolution targeted multiplex imaging in each of the cohorts. Due to the bulk analytics approach applied in untargeted proteomics, it is possible that subtle changes due to changes in localized proteins can go undetected. One such change in this current study was the differential elastin expression identified between injury groups, specifically localized to the intrapulmonary bronchioles and larger blood vessels. Targeted imaging revealed nuanced changes in ECM components, such as elastin, whereas unsupervised clustering of protein expression further expands on these observations, uncovering clear separation as well as broader patterns of protein expression and pathway activation related to the cause of injury. Although it would have been interesting to also explore COL6 and S100A using the imaging pipeline, we have not yet been able to establish these antibodies on porcine tissue, which is a general challenge when working with this model.

The activation of defense response, immune system process, cell chemotaxis, leukocyte migration, and leukocyte cell-cell adhesion pathways, among others, were significantly upregulated across all injury models. Activation of T-cell proliferation and suppression of complement activation was found to be unique to the gastric group. In contrast, monocyte chemotaxis appeared as a key driver of VILI and the activation of stress response proteins, a characteristic of LPS-mediated injury. Therefore, protein and biomarker measurement of the lung parenchyma before transplantation could be used to characterize and differentiate the often-multifactorial causes of donor lung injury, potentially predicting the ensuing risk of PGD.

Increases in exhaled particles from the airways expressed as PFR have been proposed as an early indication of ALI,^8,9^ which was also observed in this current study across all 3 donor injury causes. These increases likely stem from the disruption of alveolar endothelial and epithelial barriers, as evidenced by the loss of alveolar circularity and gross morphology seen in the immunofluorescence staining. This disruption of tight junctions is further supported by the loss of AQP5, a protein linked to tight junctions and present on the apical surface of cells.^26^ Thus, PFR could serve as a marker for the deterioration of tight junctions and the overall integrity of the alveolar barrier. Furthermore, a correlation between PFR levels and decreased PaO_2_/FiO_2_ ratios was found, further strengthening the relationship between increases in PFR and ALI. The composition of EBP in the 3 models contained many known components of BALF and differed distinctly between the 3 groups.^27^ Variations in the protein profiles of EBP and PFR have previously successfully been used to predict COVID-19 and might, therefore, be used for improved detection and classification of donor lung injury.^10^

In efforts to increase the donor lung pool, ex vivo lung perfusion (EVLP) has emerged as a key platform for evaluating and restoring lung function in the donor lung. EVLP can be leveraged for biomarker measurement as samples can be collected from the lung parenchyma, perfusate, and the ventilator outflow as EBPs. Furthermore, EVLP could be used for targeted therapies without the risk of systemic effects.^28,29^ In LTx, combining EVLP with advanced proteomic biomarkers for targeted therapies could effectively manage different ALI subphenotypes, greatly increasing the donor lung pool. A compelling illustration of this approach was recently demonstrated with the identification and subsequent silencing of metallopeptidase inhibitor 1 using small interfering RNAs to ameliorate LPS-induced ALI.^30^ Our observation of the marked upregulation of metallopeptidase inhibitor 1 in the LPS cohort corroborates this finding and introduces the possibility of using a targeted small interfering RNA cocktail or comparable gene therapy to modulate specific upregulated pathways. Additional interventions currently being considered for use during EVLP include mesenchymal stromal cells and extracellular vesicles for their immunomodulatory paracrine effects on inflammatory pathways.^31^ Recent investigations of mesenchymal stromal cells have renewed hope for repairing a damaged alveolar epithelium in cases of significant cell destruction.^32^ This cell destruction could potentially be detected by elevated cytoplasmic proteins in EBP, as observed in the gastric cohort, guiding therapeutic interventions for maximal impact. Furthermore, we found distinct cytokine profiles among groups, especially the marked increase of TNF-α and IL-10 in infection-induced ALI and IL-1β in gastric aspiration. Given the role of TNF-α role in aggravating alveolar damage, cytokine adsorption, shown to improve lung function in LPS-induced injury and reduce PGD in LTx in pigs, could be particularly beneficial for this donor lung injury subtype while modifications to IL-1β release more suitable in gastric aspiration-induced ALI.^33-35^

In conclusion, this study significantly advances the current understanding of ALI by defining the distinct differences across 3 specific etiologies of donor lung injuries. Using a comprehensive proteomic approach, including both immunofluorescent and MS techniques, our research has successfully identified and characterized unique injury-specific pathways and protein expression profiles associated with each cause. Notably, we observed consistent changes in the ECM, particularly the early downregulation of COL6, across all 3 ALI models. These insights are vital for enhancing the strategies aimed at improving the recovery and viability of damaged donor lungs. Recognizing and understanding these cause-specific variations is essential for the development of more targeted and effective treatment modalities, thereby contributing to the expansion and optimization of the donor lung pool.

## ACKNOWLEDGMENTS

Support from the Swedish National Infrastructure for Biological Mass Spectrometry is gratefully acknowledged.

## Supplementary Material


