# Supplementary material for: Unraveling Molecular and Functional Responses Across 3 Lung Injury Models to Expand the Donor Lung Pool
Source: Transplantation. 2025 Feb 19;109(7):1166–74. doi: 10.1097/TP.0000000000005353 (PMC12180699; doi:10.1097/TP.0000000000005353)
Supplement: Supplementary file 1 [file tpa-109-1166-s001.pdf]

SDC, Materials and Methods

1.1 General Methods

1.1.1 Ethical approval

The study was approved by the local Ethics Committee for Animal Research (Dnr 5.2.18-4903/16, and Dnr 5.2.18-8927/16). All animals received care according to the USA Principles of Laboratory Animal Care by the National Research Council contained in the Guide for the Care and Use of Laboratory Animals, National Academies Press (1996).

1.1.2 Animal preparation

A total of 21 Yorkshire pigs with a mean weight of 33.5 ± 1.4 kg were included in the study. The animals were stratified into three different groups (LPS, gastric, and VILI) before premedication. The animals were premedicated with intramuscular (IM) xylazine 2 mg/kg (Rompun® vet. 20 mg/ml; Bayer AG, Leverkusen, Germany), ketamine 20 mg/kg (Ketaminol® vet. 100 mg/ml; Farmaceutici Gellini S.p.A., Aprilia, Italy) and midazolam 0.5 mg/kg (Midazolam Panpharma® Oslo, Norway). General anesthesia was maintained in a supine position with continuous intravenous (IV) ketamine, fentanyl (Leptanal®, Lilly, France), and midazolam (Midazolam Panpharma®, Panpharma Nordics AS, Oslo, Norway). Isotonic Ringers Acetate (Ringer-Acetat Baxter Viaflo®, Baxter International Inc, IL, USA) was used for fluid maintenance. A secure airway was established with a tracheostomy, and the right carotid artery was cannulated with a catheter (Merit Medical Secalon-T™, Merit Medical Ireland Ltd, Galway, Ireland) for invasive monitoring together with a pulmonary artery catheter (Swan-Ganz CCOmbo V and Introflex, Edwards Lifesciences Services GmbH, Unterschleissheim, Germany) inserted in the right jugular vein. Norepinephrine

and dobutamine were used as continuous infusions to maintain a stable hemodynamic state.

1.1.3 Ventilatory settings and measurements before lung injury induction

Before lung injury measurements included 30 minutes of standard ventilation with volume-controlled ventilation (VCV), a tidal volume (Vt) of 8 ml/kg, a peak end-expiratory pressure (PEEP) 5 cmH_2O, an inspiratory-expiratory ratio (I:E) of 1:2, a respiratory rate (RR) of 20-25 breaths/min, and a fraction of inspired oxygen (FiO_2) of 0.3-0.5.

1.1.4 Infection-like induced lung injury using Lipopolysaccharide (LPS)

Mechanical ventilation in the LPS model was performed using a Maquet Servo-I ventilator (Getinge Sverige AB) according to the animal facility standards. Lipopolysaccharide (LPS) from gram-negative *Escherichia coli* (O111:B4, Sigma-Aldrich, Merck KGaA, Darmstadt, Germany) was used to induce an acute lung injury. LPS was administered intravenously through a central line as an infusion (0.5 µg/kg/min) for 1h. All LPS-treated animals developed hemodynamic instability and required a continuous infusion of norepinephrine (40 µg/ml, 0.05 – 2 µg/kg/min) (Pfizer AB, Sollentuna, Sweden) and dobutamine (2 mg/ml, 2.5 – 5 µg/kg/min) (Hameln Pharma Plus GmbH, Hameln, Germany). Fluid loss was compensated with Ringer's acetate (Baxter Medical AB, Kista, Sweden) in all animals. Mechanical ventilation followed in the VCV setting with PEEP, RR, and FiO_2 adjusted according to clinical demands as determined by anesthesiologist.

1.1.5 Ventilatory-induced lung injury with saline lavage and ventilation strategy

Mechanical ventilation in the VILI model was performed using a Maquet Servo-I ventilator (Getinge Sverige AB) according to the animal facility standards. Acute lung

injury was established with a double-hit model (1). Seven animals received 30 ml/kg of 37°C isotonic saline applied through the endotracheal tube, followed by endotracheal suctioning until a PaO_2/FiO_2 ratio below 200 mmHg was achieved. This procedure flushed out the surfactant from the alveoli to remove the surface tension, making the alveoli more prone to collapse. This was followed by approximately 120 minutes of harmful mechanical ventilation (PCV, PEEP 2 cmH_2O, inspiratory pressure 36 cmH_2O, I:E 1:2, and RR 20 bpm) to create lung injury via barotrauma and atelectotrauma. When lung injury was established, PaO_2/FiO_2 ratio below 200 mmHg, ventilation was switched to protective ventilation with VCV, Vt 6-8 ml/kg, PEEP 12 cmH_2O, I:E 1:1, RR 20 breaths/min, and FiO_2 adjusted according to clinical demands as determined by anesthesiologist.

1.1.6 Gastric aspiration-induced lung injury using gastric content

Mechanical ventilation in the gastric model was performed using a Maquet Servo-I ventilator (Getinge Sverige AB) according to the animal facility standards. Gastric contents were collected previously from four pigs deprived of food for >12 h. All animals were sedated, and an orogastric tube (36 Fr) was inserted and engaged to suction. Standardized gastric contents with pH 2 (4 mL/kg) were delivered bronchoscopically in 7 pigs. The gastric content was equally distributed between the different lung lobes. The purpose of this procedure was to mimic a clinical situation of aspiration. Mechanical ventilation followed in the VCV setting with PEEP, RR, and FiO_2 adjusted according to clinical demands as determined by anesthesiologist.

1.1.7 Particles in Exhaled Air (PExA) and Exhaled Breath Particles (EBP)

A customized PExA 2.0 device (PExA, Gothenburg, Sweden) was used in conjunction with mechanical ventilation and connected to the expiratory limb of the ventilator, as previously described (2,3). The PExA device measured particle count as a particle flow

rate (PFR, particles/minute) with the particles collected onto a membrane, measured as total accumulated mass (ng) of particles from the airways, as previously described (4). PExA measurements were recorded continuously throughout the experiment. Measured particles were in the range of 0.33-3.67 µm in diameter. Particles collected onto a membrane for biochemical analysis are referred to as exhaled breath particles (EBP). EBP samples were kept frozen at -80° C until analysis.

1.1.8 Arterial blood gases

Arterial blood gases were analyzed every 30 minutes according to clinical standards with an ABL 90 FLEX blood gas analyzer (Radiometer Medical ApS, Brønshøj, Denmark).

1.1.9 Hemodynamics

Hemodynamic parameters such as heart rate (HR), systolic blood pressure (SBP), diastolic blood pressure (DBP), mean arterial pressure (MAP), central venous pressure (CVP), cardiac output (CO), cardiac index (CI), systolic pulmonary pressure (SPP), diastolic pulmonary pressure (DPP), mean pulmonary pressure (MPP), pulmonary artery wedge pressure (PAWP), systemic vascular resistance (SVR) and pulmonary vascular resistance (PVR) were measured every 30 minutes using thermodilution with a Swan-Ganz catheter and an arterial line.

1.1.10 ARDS definition

The different ARDS stages were defined according to the Berlin definition (8) using the PaO_2/FiO_2 ratio. Mild ARDS was defined as a ratio between 201-300 mmHg, moderate as 101-200 mmHg, and severe as a ratio ≤ 100 mmHg.

1.1.11 Chest x-ray

Chest x-ray was taken before induction of lung injury and after established lung injury confirming pulmonary infiltration.

1.1.12 Cytokines and chemokines

Plasma samples were taken before lung injury and every hour until the termination of the experiment. Bronchoalveolar lavage fluid (BALF) samples were obtained before lung injury and at the end with an Ambu® aScope™ (Ambu, Helsingborg, Sweden). Plasma and BALF were analyzed with a cytokine multiplex kit, Cytokine & Chemokine 9-Plex Porcine ProcartaPlex™ Panel 1 (Thermo Fisher Scientific Cat. No. EPX090-60829-901) according to the manufacturer's instructions. The kit was analyzed using a Bioplex-200 system (BioRad Laboratories, Hercules, California). The nine cytokines in the kit included: IL-1b, IL-4, IL-6, IL-8, IL-10, IL-12, TNF-a, IFN-a and IFN-γ.

1.1.13 Histology

Lung biopsies were taken before lung injury from an inflated right lower lobe through a small right-sided thoracotomy and were also taken at the termination of the experiment through a sternotomy from inflated lower and upper lobes of both the right and left lungs. Biopsies were fixed in 10% formalin solution (Sigma Aldrich, Germany) at 4°C overnight, and paraffin-embedded following a graded series of ethanol and isopropanol (Fisher Scientific, UK). 5 μm sections on slides were stained with hematoxylin and eosin (H&E, Histolab, Sweden) and imaged with an Olympus DP23 CKX53 microscope (Olympus, Japan). Histological assessment was conducted by three independent evaluators experienced in porcine lung injury models. Scoring criteria included the quantification of alveolar and interstitial immune cells, identification of proteinaceous debris and hyaline membranes, evaluation of, measurement of alveolar wall thickening, and assessment of injury severity,

hemorrhage, and atelectasis. The scoring was adapted from a previously established methodology (5). Each feature was scored on a scale ranging from 0 to 6, and the results were presented as the mean sum of the individual characteristic scores as well as a summed score of all five characteristics.

1.1.14 Immunofluorescence staining

For immunostaining, samples were cut from paraffin blocks with a microtome at a thickness of 10um. Sections underwent de-paraffinization and antigen retrieval in citrate buffer prior to staining. After permeabilization and blocking, slices were incubated with primary antibodies (elastin, 1:200; SMA, 1:500; AQP5, 1:250) overnight at 4°C. Following washes, samples were incubated with secondary antibodies (1:1000) for 90 minutes at 4°C. Finally, samples were incubated with DAPI (1:1000) and tomato lectin (1:500) for 30 minutes at room temperature before final washes and mounting with fluoromount-G. All imaging was carried out using a Nikon confocal A1RHD.

1.1.15 Calculations and statistics

Continuous variables were reported as mean ± standard error of the mean (SEM). Hemodynamic and ventilatory changes were calculated using a Mixed-effects model (REML) with Tukey's multiple comparisons test and endpoint p-values between groups plotted. Plasma and BALF cytokine changes were first normalized to each biological replicates $\log_2$ fold change from before lung injury. Cytokine changes between groups and time points were calculated using a 2-way ANOVA with Tukey's multiple comparisons test and endpoint p-values between groups plotted. Comparison of histology scores was performed with a one-way ANOVA with Tukey's multiple comparisons. Change in PFR from before lung injury was tested with Friedman test

with Dunn´s multiple comparison test between timepoints. Correlation between PFR and PaO_2/FiO_2 values at different time points was performed with Spearman correlation and reported as r and two-tailed p-value. All statistical analyses were performed using GraphPad Prism v10.0.2. Significance was defined as: $p < 0.0001$ (****), $p < 0.001$ (***), $p < 0.01$ (**), $p < 0.05$ (*), and $p > 0.05$ (not significant).

1.2.0 Mass spectrometry and bioinformatics

1.2.1 Sample preparation and protein digestion for tissue

Biopsies were taken from the right lung following intubation as baseline and end samples and snap frozen, while samples from the lavage group were put in RNAlater prior to snap freezing. Proteins were then sourced from the pulverized tissue. These proteins were dissolved in 2% SDS (Sigma Aldrich, Darmstadt, Germany), and their protein concentration was determined using a BCA assay (Pierce, Thermo Fisher Scientific, Waltham, MA, USA). Next, 30 µg of protein underwent digestion via the S-TRAP method. The samples were first reduced with 20 mM DTT (Sigma Aldrich) at 56°C for 45 minutes, followed by a 30-minute exposure to 40 mM IAA (Sigma Aldrich) in a dark, room temperature setting. After acidifying the samples with 2.5% phosphoric acid and washing them with a buffer, they were bound to an S-Trap CO2-micro-80 column (ProTifi, Fairport, NY, USA). A dual digestion process was carried out overnight at 37°C using both lysine-C (Promega, at a 1:50 enzyme-to-protein ratio by ng) and trypsin (Promega, at a 1:50 enzyme-to-protein ratio by ng).

1.2.2 Sample preparation and protein digestion of EBP membranes

EPB membranes kept at -80°C were defrosted to room temperature and soaked in 2% SDS mixed with 50mM TEAB (TEAB, Thermo Fisher Scientific) at 37°C for 2h. The S-TRAP, as mentioned above, tissue extraction process was then applied. The

enzymatic breakdown involved using 1 µg Lys-C for 2h, then 1 µg Trypsin for 12h, followed by an added 0.45 µg Trypsin for another 2h.

1.2.3 Peptide mixing and pre-fractionation

Fractionation of tissue samples was carried out using a Waters XBridge BH130 C18 3.5 µm, 2.1 × 150 mm column on an Ultimate 3000 RS HPLC (Thermo Scientific, Waltham, MA, USA) operating at 200 µL/min. The mobile phases were solvent A: 10 mM ammonium formate pH 10, solvent B: 90% ACN, and 10% water containing 10 mM ammonium formate pH 10. Peptides were separated using the following gradient: 0 minutes 0% B; 3 minutes 0% B, 97 minutes 35% B; 98 minutes 80% B; 108 minutes 80% B. The column was operated at room temperature, and the detection wavelength was 214 nm. We collected 96 fractions at 1 minutes intervals, which were further concatenated to 48 fractions by combining 2 fractions that are 24 fractions apart, i.e., #1 and #25, #2 and #26, etc. The fractions were dried in a Speed-Vac.

1.2.4 DDA data acquisition on timsTOF Pro 2 for library generation

Fractions were resuspended in 0.1% formic acid, and peptide determination was performed in a Nanodrop system (DeNovix, Wilmington, DE, USA) before LC-MS/MS analysis. 400ng of each fraction was loaded on Evosep tips (Evosep Biosystems, Odense, Denmark) for separation with nanoflow reversed-phase chromatography with an EVOSEP ONE liquid chromatography (LC) system (Evosep Biosystems). Separation was performed with the 30 SPD method (gradient length 44 min) using a 15 cm x 150 µm Evosep column (Evosep Biosystems) packed with 1.5 µm ReproSil-Pur C18-AQ particles. The Evosep One was coupled to a timsTOF Pro 2 ion mobility mass spectrometer (Bruker, Billerica, MA, USA) operated in DDA PASEF with 10

PASEF scans per acquisition cycle and accumulation and ramp times of 100 ms each. Singly charged precursors were excluded, the ‘target value’ was set to 20,000, and dynamic exclusion was activated and set to 0.4 min. The quadrupole isolation width was set to 2 Th for m/z < 700 and 3 Th for m/z > 800. All subsequent DDA files were used to build a spectral library in Fragpipe v 18.025-28 with the following parameters: Missed cleavages=2, Min peptide length=7, Max peptide length=50, and common internal retention time peptides (CiRT) were used for spectral library retention time calibration. Uniprot UP000008227 FASTA (release 2023_01) was used as a database with reversed target sequences as decoys. The generated library consisted of 10296 protein groups in total.

1.2.5 DIA data acquisition on timsTOF Pro 2

A py_diAID method was generated by subjecting the 48 DDA fraction runs. Samples were then introduced onto Evosep tips (Evosep Biosystems) and underwent separation using the same gradient as in DDA data collection. The diaPASEF technique was employed for MS data collection. Both accumulation and ramp durations were established at 100 ms. The DIA scan settings included 25 m/z isolation windows covering a range from 247-1350 m/z and ion mobility ranges between 0.60-1.60 1/K0, with a projected cycle duration of 2.76s. Collision energy varied linearly based on mobility, ranging from 59 eV at 1/K0 = 1.6 Vs cm-2 to 20 eV at 1/K0 = 0.6 Vs cm-2.

1.2.6 Bioinformatic analysis of LC-MS/MS data

The raw tissue data from LC-MS/MS was processed using DIA-NN, v1.8.1 (6). For quantification, the Robust LC (High Precision) mode was selected, and the standard

RT-dependent normalization was employed along with the spectral library previously constructed via Fragpipe. Downstream data analysis was performed in RStudio, v2022.12.0, using R v4.3.1. For normalization and differential expression analysis, the MS-DAP software package, v1.0.5, was employed (7). Identified proteins underwent an initial filtering to retain those that appeared in at least 60% of contrasts. The data then underwent variance stabilizing normalization (VZN) and subsequent mode-between-protein normalization. The DEqMS R package was used for differential expression analysis (8). The determination of log2-fold change thresholds was achieved through bootstrapping in the MS-DAP package. Significantly differentially expressed proteins were defined as FDR-corrected p-values (q-values) less than 0.05 and bootstrapped inferred log2-fold change cut-off values. For visualization in the heatmap, MaxLFQ values were normalized using z-scoring of the data and graphically plotted using the pheatmap package, v1.0.12, employing Euclidean methods for clustering. Gene set enrichment analysis (GSEA) analysis was performed by examining all proteins found through bioinformatic analysis after the filtering and normalization steps using the clusterProfiler package v 4.8.4 (9). Annotation of ECM proteins was performed using the MatrisomeAnalyzeR R package developed by the Matrisome project (10).

EBP LC-MS/MS raw data was processed using DIA-NN in library-free mode with the Uniprot UP000008227 FASTA (release 2023_01). Downstream data analysis was performed in RStudio with the MS-DAP package. Intensity values were normalized using VZN followed by mode-between normalization. Due to the low protein amount in before lung injury samples and a high number of missing identifications in EBP data, the MSqRob2 package, which operates directly on peptide abundance values, was

used in combination with count-based peptide model and combined into a hurdle model (11,12). Due to technical issues with the LC-MS/MS instrument, four samples had to be excluded after quality control analysis. These included three samples from the LPS group and one baseline sample. String-db was used for protein-protein interaction networks and functional enrichment analysis of differentially expressed proteins in EBP (13).

2.0 Materials and Methods References

1. Lachmann B, Robertson B, Vogel J. In vivo lung lavage as an experimental model of the respiratory distress syndrome. Acta Anaesthesiol Scand. 1980 Jun;24(3):231–6.

2. Broberg E, Andreasson J, Fakhro M, Olin AC, Wagner D, Hyllén S, et al. Mechanically ventilated patients exhibit decreased particle flow in exhaled breath as compared to normal breathing patients. ERJ Open Res [Internet]. 2020 Jan 1 [cited 2020 Aug 18];6(1). Available from: https://openres.ersjournals.com/content/6/1/00198-2019

3. Broberg E, Hyllén S, Algotsson L, Wagner DE, Lindstedt S. Particle Flow Profiles From the Airways Measured by PExA Differ in Lung Transplant Recipients Who Develop Primary Graft Dysfunction. Exp Clin Transplant Off J Middle East Soc Organ Transplant. 2019 Dec;17(6):803–12.

4. Broberg E, Wlosinska M, Algotsson L, Olin AC, Wagner D, Pierre L, et al. A new way of monitoring mechanical ventilation by measurement of particle flow from the airways using Pexa method in vivo and during ex vivo lung perfusion in DCD lung transplantation. Intensive Care Med Exp. 2018 Jul 27;6(1):18.

5. Matute-Bello G, Downey G, Moore BB, Groshong SD, Matthay MA, Slutsky AS, et al. An Official American Thoracic Society Workshop Report: Features and Measurements of Experimental Acute Lung Injury in Animals. Am J Respir Cell Mol Biol. 2011 May;44(5):725–38.

6. Demichev V, Messner CB, Vernardis SI, Lilley KS, Ralser M. DIA-NN: neural networks and interference correction enable deep proteome coverage in high throughput. Nat Methods. 2020 Jan;17(1):41–4.

7. Koopmans F, Li KW, Klaassen RV, Smit AB. MS-DAP Platform for Downstream Data Analysis of Label-Free Proteomics Uncovers Optimal Workflows in Benchmark Data Sets and Increased Sensitivity in Analysis of Alzheimer's Biomarker Data. J Proteome Res. 2023 Feb 3;22(2):374–86.

8. Zhu Y, Orre LM, Zhou Tran Y, Mermelekas G, Johansson HJ, Malyutina A, et al. DEqMS: A Method for Accurate Variance Estimation in Differential Protein Expression Analysis. Mol Cell Proteomics MCP. 2020 Jun;19(6):1047–57.

9. Wu T, Hu E, Xu S, Chen M, Guo P, Dai Z, et al. clusterProfiler 4.0: A universal enrichment tool for interpreting omics data. The Innovation. 2021 Jul 1;2(3):100141.

10. Petrov PB, Considine JM, Izzi V, Naba A. Matrisome AnalyzeR – a suite of tools to annotate and quantify ECM molecules in big datasets across organisms. J Cell Sci. 2023 Sep 4;136(17):jcs261255.

11. Goeminne LJE, Sticker A, Martens L, Gevaert K, Clement L. MSqRob Takes the Missing Hurdle: Uniting Intensity- and Count-Based Proteomics. Anal Chem. 2020 May 5;92(9):6278–87.

12. Sticker A, Goeminne L, Martens L, Clement L. Robust Summarization and Inference in Proteome-wide Label-free Quantification. Mol Cell Proteomics. 2020 Jul 1;19(7):1209–19.

13. Szklarczyk D, Kirsch R, Koutrouli M, Nastou K, Mehryary F, Hachilif R, et al. The STRING database in 2023: protein-protein association networks and functional enrichment analyses for any sequenced genome of interest. Nucleic Acids Res. 2023 Jan 6;51(D1):D638–46.

Fig S1

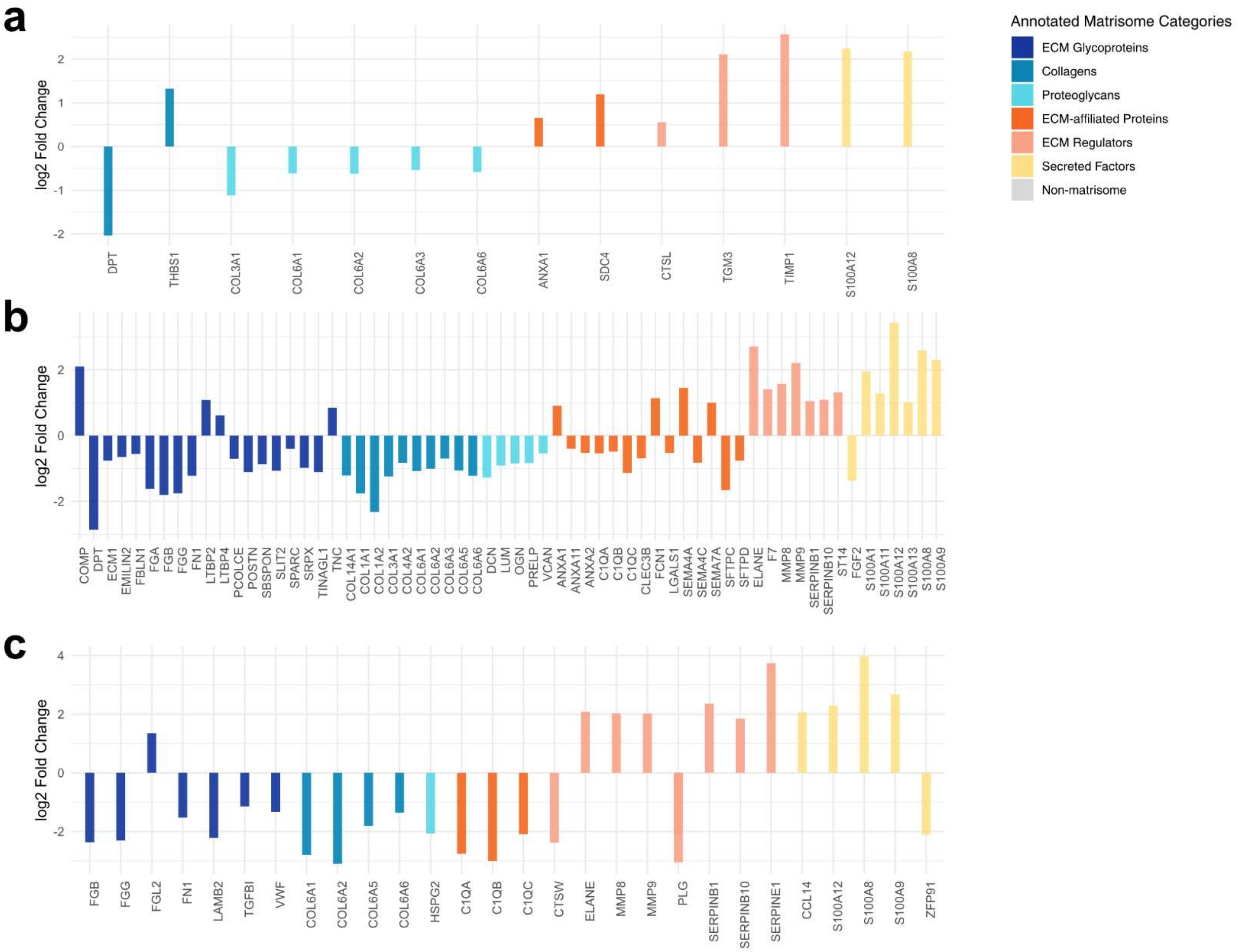

Barplots of $\log_2$ foldchange difference for significantly differentially expressed proteins annotated as belonging to the extracellular matrix for **a)** the lipopolysaccharide induced lung injury group (LPS), **b)** the gastric aspiration induced lung injury group (Gastric) and **c)** the ventilator-induced lung injury group (VILI). Data analysis was performed in R using MSqRob2 for differential expression analysis. Significantly differentially expressed proteins were defined as FDR corrected p-value of < 0.05 and boot-strapped inferred log2 foldchange cutoffs

Fig S2

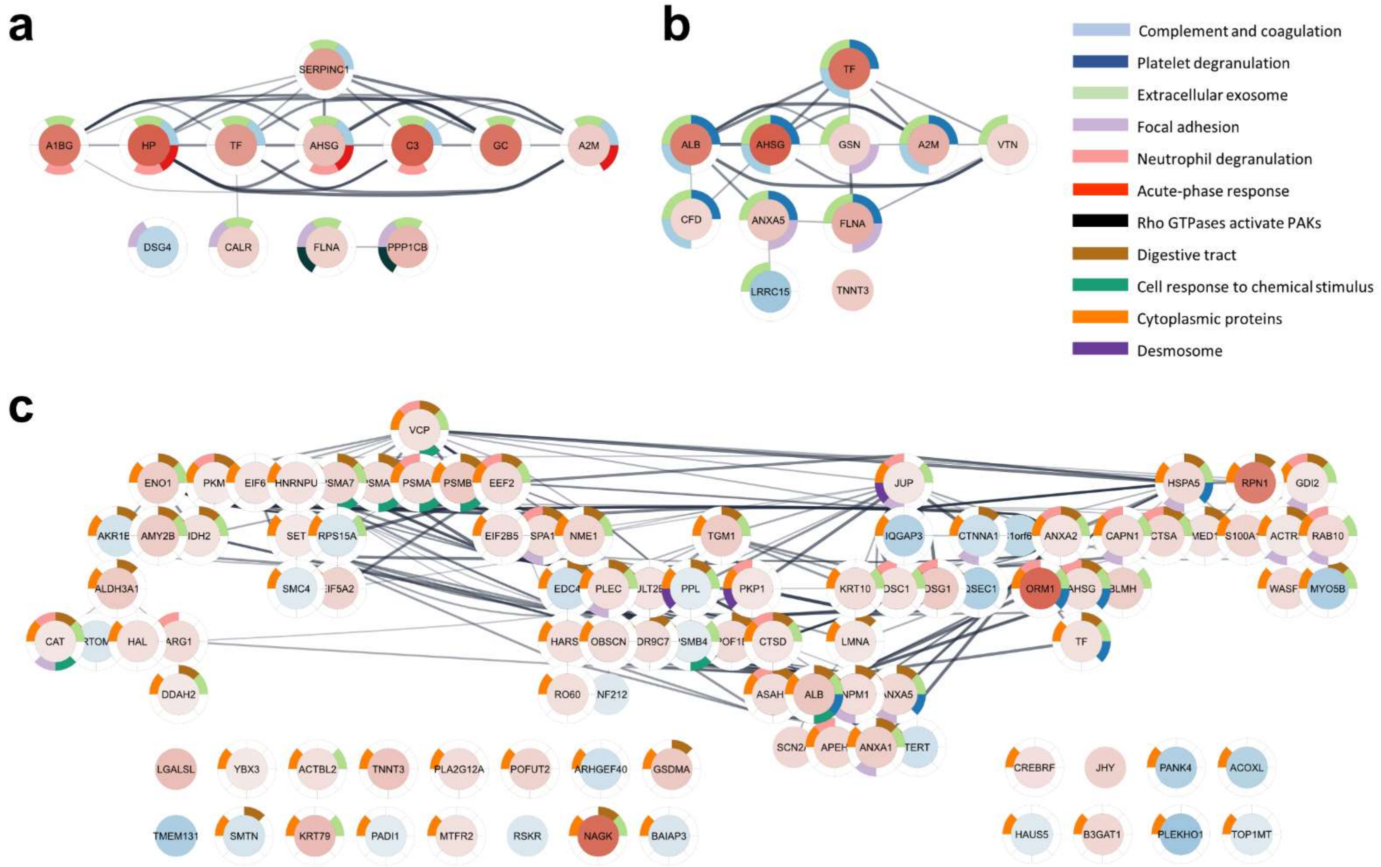

Protein-protein interaction network of significantly differentially expressed proteins from before lung injury and lung injury in **a)** the lipopolysaccharide induced lung injury group (LPS), **b)** the ventilator-induced lung injury group (VILI), and **c)** the gastric aspiration induced lung injury group (Gastric). Highly overexpressed proteins are shown in dark red and highly under-expressed in dark blue. The ring around proteins shows annotated significantly enriched pathways belonging to the protein. Data analysis was performed with string-db.org.
