# Supplementary material for: Unraveling Molecular and Functional Responses Across 3 Lung Injury Models to Expand the Donor Lung Pool
Source: Transplantation. 2025 Feb 19;109(7):1166–74. doi: 10.1097/TP.0000000000005353 (PMC12180699; doi:10.1097/TP.0000000000005353)
Supplement: Supplementary file 2 [file tpa-109-1166-s002.pdf]

Table S1. Hemodynamic measurements and oxygenation throughout the experiment in the LPS group

	Baseline	*30 min*	*60 min*	*90 min*	*120 min*	*Endpoint*
	Vitals					
Sat (%)	98.1±0.7	96.3±0.8	96.0±0.8	96.5±0.9	96±0.9	94.7±0.9
HR (bpm)	68.0±3.5	85.0±8.9	112.7±12.1	121.5±7.3	125.3±6.4	128.3±6.8
SBP (mmHg)	99.3±3.4	108.2±8.9	101.2±4.5	100.2±3.2	115.5±16.7	106.3±10.5
DBP (mmHg)	71.9±3.4	75.9±7.6	69.1±5.9	60.8±5.6	56.0±4.4	59.4±3.3
MAP (mmHg)	82.0±4.2	90.1±8.9	80.7±5.4	75.0±4.4	70.8±3.7	72.3±3.5
CVP (mmHg)	5.7±1.2	8.4±2.0	6.3±1.8	6.0±1.1	5.8±1.4	6.1±1.0
Temp (°C)	37.9±0.4	38.3±0.5	38.6±0.5	38.4±0.5	38.1±0.7	38.7±0.5
	Hemodynamics					
SPP (mmHg)	23.0±1.0	65.0±9.2	41.0±6.6	39.0±2.7	38.5±3.0	36.1±3.0
DPP (mmHg)	13.0±1.6	34.0±4.2	24.3±2.7	21.8±2.8	26.7±1.9	22.1±2.7
MPP (mmHg)	17.0±1.5	43.0±5.0	31.7±4.4	29.3±2.3	32.5±2.1	29.1±2.3
PAWP (mmHg)	9.4±1.3	14.9±3.4	11.9±3.4	12.0±3.9	9.2±1.8	11.7±3.2
CO (L/min)	4.6±0.4	5.1±0.6	6.6±0.9	7.6±1.0	6.5±1.2	7.1±0.9
SVR (DS/cm^5)	1331.0±121.0	1398.0±126.0	1092.0±174.0	765.0±90.0	1033.0±245.0	929.0±160.0
PVR (DS/cm^5)	151.4±6.5	431±76.8	307.8±77.7	273.8±42.4	282.0±50.1	255.7±38.4
	Blood gases					
pH	7.4±0.0	7.34±0.0	7.29±0.0	7.32±0.0	7.28±0.0	7.27±0.0
$PaCO_2$ (mmHg)	40.7±3.0	49.2±2.7	52.5±2.5	53.1±2.5	54.2±1.6	55.7±1.9
PaO_2 (mmHg)	260.0±13.4	171.4±28.9	157.3±17.6	149.9±23.8	133.1±16.8	104.1±6.7
Hb (g/L)	93.0±3.3	106.4±3.3	108.7±2.6	105.7±2.8	106.0±4.1	103.0±2.8
Lactate (mmol/L)	1.4±0.1	1.3±0.1	1.5±0.1	1.6±0.2	1.9±0.2	1.9±0.2
BE (mmol/L)	4.5±0.9	3.8±0.7	2.3±0.7	2.4±0.7	1.6±0.8	1.8±0.6
	Respiratory					
MV (L/min)	8.1±0.4	8.3±0.5	8.4±0.6	9.4±0.6	8.9±0.9	8.9±0.6
PIP (cmH_2O)	16.3±0.8	18.7±1.3	19.7±1.4	19.7±1.4	19.0±1.1	20.7±1.4
PEEP (cmH_2O)	4.9±0.3	5.1±0.1	5.1±0.1	5.0±0.0	5.0±0.0	5.0±0.0
Vt (mL)	396.3±13.4	391.4±15.2	384.4±13.9	395.0±13.0	381.0±9.3	393.3±11.0
Cdyn (mL/cmH_2O)	35.7±2.8	30.3±2.9	27.3±1.8	27.7±1.8	27.6±1.8	26.2±2.0
RR (breaths/min)	19.7±0.7	21.3±1.4	22.4±1.4	23.5±1.5	24.0±2.3	22.9±1.5
FiO_2	0.5±0.0	0.5±0.0	0.5±0.0	0.5±0.0	0.5±0.0	0.5±0.0
PaO_2/FiO_2(mmHg)	520.8±11.0	349.7±55.2	321.1±31.6	307.0±44.2	266.3±33.6	214.5±12.0

Abbreviations: Oxygen saturation (Sat), heart rate (HR), systolic blood pressure (SBP), diastolic blood pressure (DBP), mean arterial pressure (MAP), central venous pressure (CVP), temperature (Temp); hemodynamic variables: systolic pulmonary pressure (SPP), diastolic pulmonary pressure (DPP), mean pulmonary pressure (MPP), pulmonary artery wedge pressure (PAWP), cardiac output (CO), systemic vascular resistance (SVR), pulmonary vascular resistance (PVR); blood gas parameters: pH, partial pressure of carbon dioxide (PaCO2), partial pressure of oxygen (PaO2), hemoglobin (Hb), lactate, base excess (BE); ventilatory parameters with volume controlled ventilation: minute volume (MV), peak inspiratory pressure (PIP), positive end expiratory pressure (PEEP), tidal volume (Vt), dynamic compliance (Cdyn), respiratory rate (RR), fraction of inspired oxygen (FiO2), partial pressure of oxygen divided by fraction of inspired oxygen (PaO_2/FiO_2). Shown as mean and ± SEM.
