# Supplementary material for: Unraveling Molecular and Functional Responses Across 3 Lung Injury Models to Expand the Donor Lung Pool
Source: Transplantation. 2025 Feb 19;109(7):1166–74. doi: 10.1097/TP.0000000000005353 (PMC12180699; doi:10.1097/TP.0000000000005353)
Supplement: Supplementary file 5 [file tpa-109-1166-s005.pdf]

Table S4

LPS	Gene	Uniprot ID	Protein name	log10 q-val	Fold change
	PADI4	F1SUR0	Protein-arginine deiminase	1.46	2.07
	GMFG	A0A287AU23	Glia maturation factor	1.79	0.56
	GMIP	A0A8W4FMP1	GEM interacting protein	2.11	0.86
	THBS1	F1SS26	Thrombospondin 1	1.69	1.33
	COL6A6	A0A5G2R0Y2	Collagen type VI alpha 6 chain	1.59	-0.58
	COL6A3	A0A286ZVG7	Collagen type VI alpha 3 chain	1.55	-0.54
	TNFAIP2	A0A5G2RNN3	TNF alpha induced protein 2	1.53	0.66
	ANXA1	F1SJB5	Annexin	1.78	0.66
	RBM14	A0A287B401	RNA binding motif protein 14	1.60	-0.50
	COL6A2	I3LQ84	Collagen type VI alpha 2 chain	1.64	-0.62
	CKM	Q5XLD3	Creatine kinase M-type	1.43	-2.89
	BTF3L4	A0A287BAG8	Transcription factor BTF3	1.42	0.53
	COL6A1	I3LS72	Collagen type VI alpha 1 chain	1.69	-0.61
	TGM3	A0A480J826	Protein-glutamine gamma-glutamyltransferase E	1.86	2.11
	S100A8	C3S7K5	Protein S100	1.59	2.18
	VCAM1	F1S567	Vascular cell adhesion molecule 1	1.60	1.08
	CTSL	Q28944	Procathepsin L	1.59	0.55
	NAMPT	Q52I78	Nicotinamide phosphoribosyltransferase	1.59	0.47
	SUSD2	A0A8W4FGK0	Sushi domain containing 2	1.42	0.52
	S100A12	P80310	Protein S100-A12	1.86	2.24
	UCHL5	Q06AT3	Ubiquitin carboxyl-terminal hydrolase isozyme L5	1.44	0.54
	SDC4	Q8HZJ6	Syndecan-4	3.27	1.20
	OLR1	Q9TTK7	Oxidized low-density lipoprotein receptor 1	1.50	1.73
	RHOB	Q06AT8	RHOB	1.45	0.61
	TIMP1	P35624	Metalloproteinase inhibitor 1	4.40	2.57
	COL3A1	A0A286ZQ85	Collagen type III alpha 1 chain	1.42	-1.12
	DPT	P45846	Dermatopontin	1.42	-2.03
	PRTN3	F1S6S9	Proteinase 3	1.60	2.23
	PSMA2	A0A8W4FNV3	Proteasome 20S subunit alpha 2	1.59	0.47
	LIPG	I3LUA0	Lipoprotein lipase	1.60	1.38
	GGT5	F1RLR8	Gamma-glutamyltransferase 5	1.60	-1.65
	PVR	K7GR94	PVR cell adhesion molecule	1.60	0.97
	MERTK	A0A287A2E8	receptor protein-tyrosine kinase	1.42	-0.72
**VILI**	**Gene**	**Uniprot ID**	**Protein name**	**log10 q-val**	**Fold change**
	CYTA8	Q28987	Cystatin-A8	1.46	2.50
	LTF	Q6YT39	Lactotransferrin	1.93	2.30
	FN1	F1SS24	Fibronectin	1.41	-1.53
	ELOVL1	A0A287B122	Elongation of very long chain fatty acids protein 1	1.43	-1.97
	LAMB2	A0A287AWV5	Laminin subunit beta-2	1.55	-2.22
	HSPG2	A0A286ZHV7	Heparan sulfate proteoglycan 2	1.75	-2.07
	A0A287A/	A0A287AAG8	VWFA domain-containing protein	1.47	1.52
	FGG	A0A5G2QUU1	Fibrinogen gamma chain	1.31	-2.31
	ELANE	I3LK80	Elastase, neutrophil expressed	1.47	2.08
	CFH	A0A287BD18	Sushi domain-containing protein	1.70	-2.28
	FHL1	K7GS06	Four and a half LIM domains 1	1.56	-2.42
	CD177	F1RMW7	CD177 molecule	1.85	1.75
	ZYX	F1SRU2	Zyxin	1.46	-2.04

COL6A6	A0A5G2R0Y2	Collagen type VI alpha 6 chain	1.43	-1.36
PIGR	A0A287ADL4	Polymeric immunoglobulin receptor	1.36	-1.71
PLG	P06867	Plasminogen	1.59	-2.12
VWF	F1SL22	von Willebrand factor	1.40	-1.33
STOM	A0A287B310	Stomatin	1.43	1.46
FGB	F1RX37	Fibrinogen beta chain	1.46	-2.37
CA2	A0A287B6M0	Carbonic anhydrase	1.58	-1.38
RPS9	I3LEX0	40S ribosomal protein S9	1.38	-1.24
S100A9	K7GME6	S100 calcium binding protein A9	1.70	2.67
PGLYRP1	A0A286ZI97	Peptidoglycan-recognition protein	1.88	1.96
TGFBI	F1RHA7	Transforming growth factor beta induced	1.39	-1.14
EPB41	A0A287BAZ3	Protein 4.1	1.36	-1.14
AZU1	P80015	Azurocidin	2.02	2.81
COL6A2	I3LQ84	Collagen type VI alpha 2 chain	2.36	-3.10
MMP9	A0A287AZD5	Matrix metallopeptidase 9	1.93	2.02
SLC4A1	K7GR72	Anion exchange protein	2.02	-2.23
MRC1	A0A5G2R0H2	Mannose receptor C-type 1	1.44	-1.70
SERPINB1	F1SMW8	Serpin family B member 10	1.56	1.85
SERPINB1	F2Z5B1	Serpin family B member 1	1.85	2.36
C5	A0A287AIM8	Complement C5	1.93	-2.21
SMPD2	F1RSY4	sphingomyelin phosphodiesterase	1.43	-1.34
SLC2A3	O62787	Solute carrier family 2, facilitated glucose transporte	1.46	1.69
MPO	K7GRV6	Myeloperoxidase	1.59	1.45
ERMP1	F1SMM0	Endoplasmic reticulum metallopeptidase 1	1.46	-1.33
RPL7	A0A8W4FHN2	Ribosomal protein L7	1.40	-1.69
MMP8	F1SV69	Matrix metallopeptidase 8	1.47	2.03
GCA	A0A8W4FM64	Grancalcin	1.59	1.59
COL6A5	A0A287BK35	Collagen type VI alpha 5 chain	1.46	-1.81
LOC1007:	F1SNU4	15 kDa protein B-like	1.39	2.21
ALPL	A0A287BSC3	Alkaline phosphatase	1.53	1.87
RPL4	A0A5G2QSX6	60S ribosomal protein L4	1.47	-1.40
F1SD56	F1SD56	A-kinase anchor protein 2 C-terminal domain-contai	1.43	-1.73
CTSW	F1RU23	Cathepsin W	1.59	-2.38
COL6A1	I3LS72	Collagen type VI alpha 1 chain	2.36	-2.79
RPL6	Q2YGT9	60S ribosomal protein L6	1.70	-2.00
CRISP3	I3LVL5	Cysteine rich secretory protein 3	1.36	2.18
LPP	I3LUY1	LIM domain containing preferred translocation partn	1.31	-2.13
C4BPA	F1S0J2	Sushi domain-containing protein	1.92	-3.79
EPB42	F1SI48	Erythrocyte membrane protein band 4.2	1.68	-2.07
A0A8W4F	A0A8W4FLE1	60S ribosomal protein L13a	1.36	-2.07
FOLR1	A0A287BLX2	Folate receptor alpha	1.47	1.66
LOC1005	I3LS74	Antileukoproteinase	1.56	1.63
A0A287AI	A0A287AIC7	UPAR/Ly6 domain-containing protein	2.02	3.07
S100A8	C3S7K5	Protein S100	1.57	3.96
PALLD	A0A287BPI5	Palladin isoform X4	1.56	-1.37
FGL2	F6Q194	Fibrinogen like 2	1.46	1.35
PRPF8	F1RHH8	Pre-mRNA processing factor 8	1.39	-1.75
PMAP37	P49932	Antibacterial peptide PMAP-37	1.31	2.71
ANK1	F1SE30	Ankyrin 1	1.93	-1.99
RETN	Q6QR67	Resistin	1.68	2.91

	BLVRB	I3LQH7	Biliverdin reductase B	1.44	-1.19
	PRDX4	F1SQ01	Peroxiredoxin 4	1.56	-2.84
	LOC1006	I3LMQ4	START domain-containing protein	1.31	-1.75
	TM9SF4	A0A287BSC9	Transmembrane 9 superfamily member	1.47	-1.45
	ZFP91	A0A287AQ40	ZFP91 zinc finger protein, atypical E3 ubiquitin ligase	1.43	-2.10
	S100A12	P80310	Protein S100-A12	1.57	2.30
	ACP3	F1SPF0	Acid phosphatase 3	1.31	1.92
	AGO2	A0A5G2QC41	Protein argonaute-2	1.36	-1.75
	OLR1	Q9TTK7	Oxidized low-density lipoprotein receptor 1	2.02	2.58
	PLG	A0A5G2QXR3	Plasminogen	1.80	-3.05
	ALG10	I3LNP0	Dol-P-Glc:Glc(2)Man(9)GlcNAc(2)-PP-Dol alpha-1,2	1.31	-1.94
	NPG1	A0A5G2QNY2	Protegrin-1	1.45	2.57
	C1QB	A0A5G2QNN2	Complement C1q B chain	1.59	-3.01
	RPL15	A0A286ZL65	Ribosomal protein L15	1.70	-2.65
	PTI	F1SD24	BPTI/Kunitz inhibitor domain-containing protein	1.70	2.90
	TCN1	A0A287A0W9	Transcobalamin-1	2.02	2.76
	SLC34A2	F1S5A6	Sodium-dependent phosphate transport protein 2B i	1.51	-1.44
	RHAG	F1RPZ7	Rh associated glycoprotein	1.42	-2.31
	C1QC	A0A286ZSJ7	Complement C1q C chain	1.46	-2.09
	RNF123	A0A286ZKD3	Ring finger protein 123	1.43	-1.53
	SLC35B2	F1RQU1	Solute carrier family 35 member B2	1.47	-1.64
	PRTN3	F1S6S9	Proteinase 3	1.93	2.93
	C1QA	Q69DL0	Complement C1q subcomponent subunit A	1.55	-2.76
	A0A5G2Q	A0A5G2QDH1	Cathelicidin antimicrobial peptide preproprotein	1.93	2.75
	H1-0	A0A287BQW3	H1.0 linker histone	1.34	-1.69
	CKMT1A	F1SI77	creatine kinase	1.36	-1.43
	APOR	Q03472	Apolipoprotein R	1.59	-3.61
	CCL14	A0A287BE47	C-C motif chemokine	1.36	2.07
	IFNGR1	F1S692	Interferon gamma receptor 1	1.56	2.39
	PDGFRA	A0A287AYB4	Platelet-derived growth factor receptor alpha	1.40	2.17
	SERPINE1	P79335	Plasminogen activator inhibitor 1	1.47	3.74
	LCN2	A0A287BJ65	Lipocalin 2	2.36	2.83
	PLAUR	A0A287B9N1	Plasminogen activator, urokinase receptor	1.43	1.50
	LOC1102	A0A5G2QTP8	Ig-like domain-containing protein	1.53	2.61
	FCER1G	Q9XSZ6	High affinity immunoglobulin epsilon receptor subun	1.40	-2.51
	MGST3	Q2EN77	Microsomal glutathione S-transferase 3	1.40	-1.50
	CCR1	Q6YST0	C-C motif chemokine receptor 1	1.36	2.08
**Gastric**	**Gene**	**Uniprot ID**	**Protein name**	**.og10 q-value**	**Fold change**
	ABCB6	F1SR85	ATP-binding cassette sub-family B member 6	1.76	-0.54
	ABCC3	A0A5G2R2F8	ABC-type glutathione-S-conjugate transporter	1.80	0.38
	ABRACL	F1S6Z3	Costars family protein ABRACL	2.00	2.11
	ACADS	A0A287AP64	Acyl-CoA dehydrogenase short chain	2.33	-0.92
	ACSL1	A0A287AIP7	Long-chain-fatty-acid--CoA ligase	1.86	0.40
	ACTB	Q6QAQ1	Actin, cytoplasmic 1	1.63	-0.61
	ACTG2	A0A287BGY0	Actin gamma 2, smooth muscle	1.75	-0.70
	ACTR1B	F1STD5	Actin related protein 1B	1.39	0.49
	ADA2	P58780	Adenosine deaminase 2	2.33	0.79
	ADCY3	F1SDL2	Adenylate cyclase 3	1.55	0.51
	ADD1	A0A286ZR45	Adducin 1	2.69	-0.50

ADD2	I3L5B0	Adducin 2	2.66	-1.27
ADGRE5	K7GMF6	Adhesion G protein-coupled receptor E5	1.53	0.37
ADPGK	A0A5G2QK01	ADP dependent glucokinase	1.47	0.51
AFMID	A0A287A0R5	Kynurenine formamidase	1.51	0.36
AGFG1	F1SNM7	Arf-GAP domain and FG repeat-containing protein 1 i	1.44	-0.91
AHNAK2	A0A286ZXM0	PDZ domain-containing protein	1.75	-1.03
AIDA	A0A5G2R9E4	Axin interactor, dorsalization associated	1.35	0.43
AK1	P00571	Adenylate kinase isoenzyme 1	1.68	-0.98
AKAP12	A0A287A3A3	A-kinase anchoring protein 12	2.46	-0.76
AKAP6	I3L6S3	A-kinase anchor protein 6	1.86	-1.13
AKR1C1	A0A480YJR0	Aldo-keto reductase family 1 member C1	1.57	1.54
ALAD	A0A287BGW4	Delta-aminolevulinic acid dehydratase	1.45	-0.59
ALB	P08835	Albumin	1.57	-0.89
ALB	A0A287BAY9	Albumin	1.65	-1.06
ALG2	F1SSE6	Alpha-1,3/1,6-mannosyltransferase ALG2	2.00	0.46
ALOX15	P16469	Polyunsaturated fatty acid lipoxygenase ALOX15	1.76	2.16
ALYREF	A0A5G2R218	Aly/REF export factor	2.23	-1.02
AMPD2	A0A287ALM1	AMP deaminase	1.65	0.55
AMPD3	A0A287A3U3	AMP deaminase	1.52	0.74
ANK1	F1SE30	Ankyrin 1	2.38	-1.33
ANP32E	A0A286ZL69	Acidic leucine-rich nuclear phosphoprotein 32 family	3.26	1.35
ANTXR2	K9J4T3	Anthrax toxin receptor	2.19	0.69
ANXA1	F1SJB5	Annexin	2.39	0.91
ANXA11	A0A5K1UZZ4	Annexin	1.61	-0.40
ANXA2	A0A286ZJV6	Annexin	2.17	-0.52
APBB1IP	A0A286ZVU8	Amyloid beta protein binding family B member 1 inte	1.42	0.56
APMAP	A0A287A6K7	Adipocyte plasma membrane-associated protein	1.61	0.48
APOR	Q03472	Apolipoprotein R	2.57	-1.38
AQP1	Q6PQZ1	Aquaporin-1	1.85	-1.56
AQP3	A9Y006	Aquaporin-3	1.55	1.13
ARFGAP1	A0A286ZJ05	ADP ribosylation factor GTPase activating protein 1	1.71	-0.78
ARFRP1	A0A287AP37	ADP ribosylation factor related protein 1	1.89	0.86
ARGLU1	A0A5G2RFB3	Arginine and glutamate rich 1	1.50	0.66
ARHGAP1	A0A287AV22	Rho GTPase activating protein 12	1.66	-0.85
ARHGAP1	A0A287BKM4	Rho GTPase activating protein 15	1.61	1.01
ARHGAP6	K7GN21	Rho GTPase activating protein 6	1.44	-0.76
ARHGDIB	A0A287B0I3	Rho GDP dissociation inhibitor beta	1.47	0.50
ARHGEF6	A0A287AH65	Rac/Cdc42 guanine nucleotide exchange factor 6	2.06	0.49
ARL2BP	A0A5G2QXD5	ADP-ribosylation factor-like protein 2-binding protein	1.31	0.75
ARPIN	A0A287B5T1	Arpin	1.78	-0.41
ARRB2	A0A5G2QR25	Arrestin beta 2	1.73	0.71
ARSD	I3LM95	Arylsulfatase D	1.83	-0.69
ATL2	A0A287AWD2	Atlastin GTPase 2	1.97	0.57
ATOX1	I3L7P7	Copper transport protein ATOX1	1.46	0.56
ATP2A3	O77696	Sarcoplasmic/endoplasmic reticulum calcium ATPa	1.36	0.41
ATP5F1D	A0A5G2RN51	ATP synthase F1 subunit delta	1.44	0.62
ATP5PO	Q2EN81	ATP synthase subunit O, mitochondrial	2.06	-0.68
ATP7A	A0A5K1U0J0	P-type Cu(+) transporter	1.39	-0.68
ATP8A1	A0A5G2RAC3	Phospholipid-transporting ATPase	1.94	0.84
ATRX	F1RPI9	DNA helicase	1.42	1.11

ATXN2	A0A286ZI01	Ataxin 2	2.41	-0.97
AUP1	F1SNV5	AUP1 lipid droplet regulating VLDL assembly factor	1.76	-0.83
AZU1	P80015	Azurocidin	2.00	2.26
BAG5	K9IVC2	BAG cochaperone 5	2.18	0.37
BANF1	F1RU33	BAF nuclear assembly factor 1	1.64	0.67
BAZ1B	I3LCE6	Bromodomain adjacent to zinc finger domain 1B	2.20	0.48
BCAP31	F1S2A8	B-cell receptor-associated protein	2.18	-0.41
BCL2L15	A0A8W4FKU4	BCL2 like 15	1.61	1.60
BIN2	I3L7Y9	Bridging integrator 2	1.43	0.62
BLVRB	I3LQH7	Biliverdin reductase B	2.11	-1.13
BMP2K	I3LT15	BMP2 inducible kinase	1.67	-0.58
BMPR2	F1SI16	receptor protein serine/threonine kinase	1.62	0.76
BORCS6	I3L825	BLOC-1-related complex subunit 6	1.75	-0.44
BPGM	A0A286ZQ31	Phosphoglycerate mutase	1.62	-0.83
C1QA	Q69DL0	Complement C1q subcomponent subunit A	1.44	-0.54
C1QB	A0A5G2QNN2	Complement C1q B chain	1.44	-0.49
C1QC	A0A286ZSJ7	Complement C1q C chain	2.41	-1.14
C2H5ORF	A0A286ZVC5	Chromosome 2 C5orf24 homolog	2.26	-1.91
C6	A0A5G2R8T9	Complement component C6	1.38	-0.69
C8B	A0A287AT36	Complement C8 beta chain	1.47	-0.94
C8G	A0A287AFQ4	Complement C8 gamma chain	1.33	-0.94
CA1	A0A287AI92	Carbonic anhydrase	2.66	-1.73
CA2	A0A287B6M0	Carbonic anhydrase	2.36	-1.35
CA3	Q5S1S4	Carbonic anhydrase 3	2.19	-3.57
CADM1	A0A5G2QPJ4	Cell adhesion molecule 1	1.80	0.41
CALHM5	A0A286ZV34	Calcium homeostasis modulator family member 5	1.91	0.66
CALM3	A0A5G2QWK6	Calmodulin 3	2.06	1.77
CARD9	A0A0H5AX08	CARD9	1.99	1.11
CASP7	A0A287AGZ2	Caspase 7	1.93	0.53
CBR2	A0A287BT95	Carbonyl reductase [NADPH] 2	1.76	-0.83
CBX5	F2Z5T3	Chromobox 5	2.21	0.78
CCDC43	F1RQZ4	Coiled-coil domain-containing protein 43	1.30	0.88
CCDC6	F1RG04	Coiled-coil domain containing 6	1.37	-0.39
CCDC9B	F1SS33	Coiled-coil domain containing 9B	1.81	-1.56
CCR1	Q6YST0	C-C motif chemokine receptor 1	2.23	1.46
CCT3	Q29068	T-complex protein 1 subunit gamma (Fragment)	1.76	0.70
CD177	F1RMW7	CD177 molecule	2.39	3.07
CD180	Q7YRL4	CD180 antigen	1.31	0.66
CD2	K7GSI1	T-cell surface antigen CD2	1.61	-0.74
CD2AP	A0A8X9AEN0	CD2 associated protein	1.38	-0.41
CD302	A8WH75	CD302 antigen	1.40	0.71
CD53	F1S628	Tetraspanin	2.61	1.45
CD55	A0A287B6Q2	Sushi domain-containing protein	2.17	0.66
CD68_TV1	K9J6L9	Macrosialin isoform A	1.70	-0.89
CD82	A0A8W4F916	Tetraspanin	1.50	0.48
CDA	I3LAH9	Cytidine deaminase	1.59	1.10
CDC42EP	F1RV35	CDC42 effector protein 4	2.02	-0.78
CDIPT	D0G6R6	CDP-diacylglycerol--inositol 3-phosphatidyltransfera	2.24	-0.86
CELF1	A0A287B4N3	CUGBP Elav-like family member 1	1.57	0.54
CFH	A0A287BD18	Sushi domain-containing protein	1.66	-0.83

CGGBP1	A0A287ARL7	CGG triplet repeat-binding protein 1	2.46	-1.72
CHCHD2	F1RIU9	Coiled-coil-helix-coiled-coil-helix domain containing	1.97	-0.71
CHD2	A0A287A4M4	DNA helicase	1.74	0.60
CHMP5	F1SE87	Charged multivesicular body protein 5	1.31	-0.58
CIB1	F1SJX5	Calcium and integrin binding 1	2.34	1.94
CKM	Q5XLD3	Creatine kinase M-type	2.45	-3.78
CLEC3B	F1SRC8	C-type lectin domain family 3 member B	1.50	-0.69
CMLKR1	B1PHQ8	Chemerin-like receptor 1	1.78	0.98
CNN2	Q08094	Calponin-2 (Fragment)	1.38	-0.50
CNNM4	F1STC6	Metal transporter	1.47	0.69
CNPY2	F1SLY7	Canopy FGF signaling regulator 2	2.23	0.53
COBLL1	A0A287A580	Cordon-bleu WH2 repeat protein like 1	2.43	-0.63
COL14A1	A0A480I6I0	Collagen alpha-1(XIV) chain	1.87	-1.21
COL1A1	A0A287A1S6	Collagen alpha-1(I) chain preproprotein	2.32	-1.76
COL1A2	A0A8W4FM91	Collagen type I alpha 2 chain	2.13	-2.32
COL3A1	A0A286ZQ85	Collagen type III alpha 1 chain	2.00	-1.24
COL4A2	F1RLL9	Collagen type IV alpha 2 chain	1.46	-0.83
COL6A1	I3LS72	Collagen type VI alpha 1 chain	1.97	-1.08
COL6A2	I3LQ84	Collagen type VI alpha 2 chain	1.80	-1.00
COL6A3	A0A286ZVG7	Collagen type VI alpha 3 chain	1.53	-0.70
COL6A5	A0A287BK35	Collagen type VI alpha 5 chain	1.57	-1.06
COL6A6	A0A5G2R0Y2	Collagen type VI alpha 6 chain	2.13	-1.22
COMMD2	F1SJN9	COMM domain containing 2	1.36	0.45
COMMD3	F1RVH4	COMM domain-containing protein 3	2.43	1.37
COMMD5	A0A287A8L0	COMM domain-containing protein 5	1.46	0.43
COMMD9	A0A5G2QQT4	COMM domain containing 9	1.48	0.96
COMP	F1S902	Cartilage oligomeric matrix protein	2.35	2.10
COMT	F1RHN3	Catechol O-methyltransferase	1.44	0.42
COPE	F1S7E1	Coatomer subunit epsilon	1.66	0.73
COPS8	F1SM07	COP9 signalosome complex subunit 8	1.58	-0.44
COPZ1	A0A5G2QVD5	Coatomer subunit zeta	1.39	0.73
COPZ2	A0A481CG38	Coatomer subunit zeta	1.53	-0.47
CORO1A	A0A287BDS7	Coronin	1.69	0.78
COX6A1	A0A287BGN0	Cytochrome c oxidase subunit	1.71	0.77
COX6C	A1XQT2	Cytochrome c oxidase subunit 6C	1.63	-0.86
COX7A1	Q8SPJ9	Cytochrome c oxidase subunit 7A1, mitochondrial	1.65	-0.60
CPM	A0A287AW11	Carboxypeptidase M	1.44	0.64
CPSF7	F1RKQ8	Cleavage and polyadenylation specific factor 7	1.76	-0.65
CRISP3	I3LVL5	Cysteine rich secretory protein 3	2.03	2.19
CSF2RA	A0A287BFJ1	Fibronectin type-III domain-containing protein	1.67	1.16
CSNK2B	P67872	Casein kinase II subunit beta	1.50	0.64
CTDSP1	A0A287AGC6	protein-serine/threonine phosphatase	1.76	0.76
CTNNBL1	F1SEK6	Catenin beta like 1	1.61	0.61
CTTN	A0A5G2QWJ7	Cortactin	1.54	-0.50
CUTA	F1RZR6	CutA divalent cation tolerance homolog	1.80	1.35
CXCR2	F1SS04	C-X-C chemokine receptor type 2	2.02	2.16
CYBB	K7GKS3	Cytochrome b-245 beta chain	1.93	0.76
CYCS	P62895	Cytochrome c	2.18	0.44
CYFIP2	A0A287AVV3	Cytoplasmic FMR1-interacting protein	1.89	0.68
CYP2S1	M3VK37	Cytochrome P450	1.69	-0.56

CYRIB	A0A287B0D0	CYFIP related Rac1 interactor B	1.94	0.54
CYTH1	A0A5G2RLR5	Cytohesin 1	1.41	0.69
DAGLB	A0A8W4F817	sn-1-specific diacylglycerol lipase	1.52	0.94
DBI	P12026	Acyl-CoA-binding protein	1.42	0.63
DBNL	F1ST81	Drebrin like	1.45	-0.38
DCAF1	A0A287AWA9	DDB1 and CUL4 associated factor 1	1.66	0.43
DCN	Q9XSD9	Decorin	2.04	-1.28
DCP1A	I3LHS8	mRNA-decapping enzyme 1A	2.14	-0.66
DCTN2	A0A5G2QD80	Dynactin subunit 2	2.33	0.78
DCTN3	F1SEC0	Dynactin subunit 3	1.42	0.77
DCTN4	A0A4X1TB62	Dynactin subunit 4	1.75	-0.36
DDX39A	A0A287BN69	RNA helicase	1.70	0.68
DEK	A0A5G2QII0	DEK proto-onco	2.66	0.56
DERL1	A0A286ZNK2	Derlin	1.99	-0.96
DHX36	M3VK01	RNA helicase	1.68	0.40
DIDO1	A0A287AIZ5	Death inducer-obliterator 1	1.86	-1.18
DIO3	Q6QN11	Thyroxine 5-deiodinase	1.37	5.66
DLST	Q9N0F1	Dihydrolipoyllysine-residue succinyltransferase com	3.26	-0.77
DMTN	F1RMC1	Dematin actin binding protein	1.75	-1.16
DNAJB4	F1S9T0	DnaJ heat shock protein family (Hsp40) member B4	1.43	-0.41
DNAJC25	F1SNC5	DnaJ homolog subfamily C member 25	1.61	-1.04
DNAJC9	I3LRH1	DnaJ heat shock protein family (Hsp40) member C9	1.68	0.41
DNTTIP1	F1SC86	Deoxynucleotidyltransferase terminal-interacting pr	1.97	0.55
DOCK2	K9J6I8	Dedicator of cytokinesis 2	1.39	0.38
DOCK5	F1RJU3	Dedicator of cytokinesis 5	1.44	1.50
DOK3	A0A5G2QL54	Docking protein 3	1.46	0.58
DOP1B	F1SGX3	DOP1 leucine zipper like protein B	2.33	1.58
DPH5	F1S569	diphthine methyl ester synthase	1.48	-0.64
DPT	P45846	Dermatopontin	2.46	-2.87
DTNA	A0A287ADQ2	Dystrobrevin	2.21	-0.90
DUOX2	Q8HZK2	Dual oxidase 2	1.56	-0.54
DYNLL1	D9U8D1	Dynein light chain	1.89	0.44
ECM1	I3LC64	Extracellular matrix protein 1	1.76	-0.76
EEF1A1	A0A286ZUI3	Eukaryotic translation elongation factor 1 alpha 1	1.36	-0.35
EEF1D	I3LT08	Eukaryotic translation elongation factor 1 delta	1.41	0.44
EI24	A0A8W4FH42	EI24 autophagy associated transmembrane protein	1.33	-0.52
EIF2B2	A0A5G2QN40	Eukaryotic translation initiation factor 2B subunit bet	1.45	0.48
EIF2B3	I3LN50	Eukaryotic translation initiation factor 2B subunit gai	1.63	0.41
EIF5B	F1STE8	Eukaryotic translation initiation factor 5B	2.33	0.50
ELANE	I3LK80	Elastase, neutrophil expressed	2.39	2.71
ELF1	A0A5K1UL10	E74 like ETS transcription factor 1	1.90	-0.86
ELOC	A0A5G2QXY8	Elongin-C	1.63	0.44
EMD	A0A0U2ETD0	Emerin	2.56	-1.09
EMILIN2	F1SBC8	Elastin microfibril interfacer 2	1.61	-0.65
EMP1	A0A287B0D8	Epithelial membrane protein 1	1.81	-1.19
ENAH	A0A287AF78	ENAH actin regulator	3.00	-0.86
ENO2	I3LCN1	phosphopyruvate hydratase	2.36	0.66
ENO3	A0A5G2R7R6	phosphopyruvate hydratase	1.44	-1.18
ENOPH1	F1RVD2	Enolase-phosphatase E1	3.10	0.71
ENY2	A0A286ZK95	Transcription and mRNA export factor ENY2	1.74	0.96

EPB41	A0A287BAZ3	Protein 4.1	2.35	-0.68
EPB42	F1SI48	Erythrocyte membrane protein band 4.2	2.36	-1.37
EPDR1	I3LRH5	Mammalian ependymin-related protein 1	1.45	-0.58
EPHA1	F1SRW1	receptor protein-tyrosine kinase	1.53	-1.44
EPN1	A0A286ZY48	Epsin 1	2.23	-0.47
EPX	F1RSB4	Eosinophil peroxidase	2.39	-2.26
ERH	F2Z5J5	Enhancer of rudimentary homolog	1.54	0.81
EVL	A0A8W4FE99	Enah/Vasp-like	2.31	-0.77
EXOC4	A0A5G2QVV2	Exocyst complex component Sec8	1.81	0.54
F7	F1RN40	Coagulation factor VII	1.98	1.41
FABP5	A0A286ZZ87	Fatty acid binding protein 5	2.23	-1.03
FAM107A	A0A5G2R7E3	ILEI/PANDER domain-containing protein	1.39	-0.62
FAM151B	I3LUI5	Family with sequence similarity 151 member B	1.70	0.56
FBLN1	A0A287AL89	Fibulin-1	1.54	-0.56
FBXO30	A0A287ASK2	F-box protein 30	1.52	0.80
FCHO2	A0A287ANV0	F-BAR domain only protein 2	1.53	0.36
FCN1	Q29042	Ficolin-1	1.59	1.14
FERMT3	F1RQ01	FERM domain containing kindlin 3	1.58	0.41
FES	F1RMJ0	Tyrosine-protein kinase	1.31	0.37
FGA	F1RX36	Fibrinogen alpha chain	3.26	-1.62
FGB	F1RX37	Fibrinogen beta chain	3.09	-1.81
FGD3	I3L837	FYVE, RhoGEF and PH domain containing 3	1.39	0.68
FGF2	A0A287BGK8	Fibroblast growth factor	2.04	-1.37
FGG	A0A5G2QUU1	Fibrinogen gamma chain	3.26	-1.76
FGR	A0A287B9K1	Tyrosine-protein kinase	1.74	0.85
FHIP1B	A0A5K1VS40	FHF complex subunit HOOK interacting protein 1B	1.40	-0.46
FHL1	K7GS06	Four and a half LIM domains 1	1.45	-0.54
FHL3	F1SV27	Four and a half LIM domains 3	1.55	-0.43
FIBP	F1RU24	Acidic fibroblast growth factor intracellular-binding p	1.33	0.46
FIP1L1	A0A287ACH9	Pre-mRNA 3'-end-processing factor FIP1	1.55	-0.64
FKBP1A	Q2VTP6	peptidylprolyl isomerase	2.61	-0.67
FKBP5	A0A8W4FCG7	peptidylprolyl isomerase	1.65	0.38
FLII	A0A480HZP7	FLII actin remodeling protein	1.67	0.37
FLT1	A0A5G2QFF1	receptor protein-tyrosine kinase	1.67	0.67
FMNL1	A0A480LCB3	Formin like 1	1.44	0.45
FMO1	P16549	Flavin-containing monooxygenase 1	1.44	0.41
FMO3	I3LS94	Dimethylaniline monooxygenase [N-oxide-forming] 3	1.70	1.03
FN1	F1SS24	Fibronectin	2.70	-1.22
FNBP1L	F1S535	Formin-binding protein 1-like	1.93	0.58
FOLR1	A0A287BLX2	Folate receptor alpha	2.55	2.32
FUBP1	F1S9S9	Far upstream element binding protein 1	1.58	-0.36
FUBP3	A0A5G2QHD9	Far upstream element binding protein 3	1.44	-0.46
FUS	K7GSY7	FUS RNA binding protein	1.54	-0.80
FYTTD1	A0A5G2QUB4	UAP56-interacting factor	2.10	-0.88
GALNS	Q8WNQ7	N-acetylgalactosamine-6-sulfatase	1.67	0.59
GAMT	I3LIA4	guanidinoacetate N-methyltransferase	1.57	0.38
GATAD2A	A0A5G2R2J4	GATA zinc finger domain containing 2A	1.93	-0.69
GATAD2B	A0A287AQH0	GATA zinc finger domain containing 2B	2.66	-1.25
GCA	A0A8W4FM64	Grancalcin	2.19	1.31
GET4	A0A5G2QEV8	Guided entry of tail-anchored proteins factor 4	1.44	0.39

GFPT1	A0A286ZUW0	glutamine--fructose-6-phosphate transaminase (iso	1.64	0.42
GGH	A0A286ZMB2	folate gamma-glutamyl hydrolase	2.03	0.70
GLRX3	A0A5G2RBM6	Glutaredoxin 3	1.94	0.37
GLUL	P46410	Glutamine synthetase	1.80	0.41
GMFB	I3L5V7	Glia maturation factor	1.65	0.68
GNAI2	Q06AS6	G protein subunit alpha i2	2.21	0.40
GNG12	B9TRW9	Guanine nucleotide-binding protein subunit gamma	1.59	0.62
GORASP1	A0A5G2QS11	Golgi reassembly stacking protein 1	1.88	0.66
GORASP2	A0A5G2QDE7	Golgi reassembly stacking protein 2	1.70	-0.46
GPCPD1	A0A287B7N9	Glycerophosphocholine phosphodiesterase 1	1.87	0.51
GPD1	A0A286ZI55	Glycerol-3-phosphate dehydrogenase [NAD(+)]	2.00	-3.28
GPX4	P36968	Phospholipid hydroperoxide glutathione peroxidase	1.35	-0.42
GRAP	F1SB17	GRB2 related adaptor protein	1.64	0.84
GRK6	A0A287AM21	G protein-coupled receptor kinase	1.68	0.90
GRM1	A0A287AUS4	Glutamate metabotropic receptor 1	1.84	-1.14
GTF2F2	A0A286ZZT9	General transcription factor IIF subunit 2	1.67	-0.47
GUCY1B1	I3LMI1	guanylate cyclase	2.11	0.45
H1-2	A0A8W4FCQ5	Histone H2B	1.47	-0.59
H3C1	A0A287BKF7	Histone H2A/H2B/H3 domain-containing protein	1.37	-0.56
HADH	P00348	Hydroxyacyl-coenzyme A dehydrogenase, mitochonc	1.59	-0.39
HBS1L	A0A5G2QH78	HBS1-like protein N-terminal domain-containing pro	1.67	-0.93
HCK	A0A287BSA9	Tyrosine-protein kinase	1.84	0.79
HIRA	F1RK64	Protein HIRA	1.39	1.13
HK3	A0A287AE54	hexokinase	1.49	1.07
HMG20A	A0A480TTD5	High mobility group 20A	1.46	0.57
HMGB1	A0A8W4F6P8	High mobility group protein B1	1.64	0.40
HMGB2	P17741	High mobility group protein B2	2.41	0.93
HNRNPC	I3LK71	Heterogeneous nuclear ribonucleoproteins C1/C2 is	1.95	-0.55
HNRNPH3	A0A287A6R2	Heteroous nuclear ribonucleoprotein H3	1.62	-0.41
HNRNPK	I3LQS0	Heterogeneous nuclear ribonucleoprotein K	1.54	-0.47
HNRNPM	F1SA50	Heteroous nuclear ribonucleoprotein M	2.41	-0.60
HPCAL1	Q06AT0	Hippocalcin-like protein 1	2.23	1.42
HSPBP1	A0A5G2QLG3	HSPA (Hsp70) binding protein 1	2.23	0.42
HSPE1	F1SMZ6	10 kDa heat shock protein, mitochondrial	2.36	-1.10
ICAM3	K7GR08	Intercellular adhesion molecule 3	1.93	2.18
INPP5D	A0A287ABR0	phosphatidylinositol-3,4,5-trisphosphate 5-phospha	1.92	0.44
INTS5	I3LMH3	Integrator complex subunit 5	2.22	1.18
IPO7	A0A287B2B4	Importin 7	1.77	0.47
IPO9	A0A287AKZ8	Importin 9	1.65	0.47
IRAK3	A0A5G2QF58	Interleukin 1 receptor associated kinase 3	1.48	0.46
IRF2BP2	A0A287AKG8	Interferon regulatory factor 2 binding protein 2	2.08	-0.75
IRF2BPL	A0A8W4FEM4	Interferon regulatory factor 2 binding protein like	1.95	-0.65
ITFG1	A0A287A704	Integrin alpha FG-GAP repeat containing 1	1.59	0.46
ITGB2	A0A287AK86	Integrin beta	1.65	0.62
ITPRIP	F1S5N6	Inositol 1,4,5-trisphosphate receptor interacting pro	1.70	0.77
ITSN1	A0A5G2QFN5	Intersectin 1	1.70	-0.40
KCTD12	A0A8W4FMQ4	Potassium channel tetramerization domain containi	1.55	0.38
KHDRBS1	I3LP11	KH RNA binding domain containing, signal transduct	1.46	-0.41
KIF3A	A0A5G2RB39	Kinesin-like protein	1.55	1.24
KRT3	A0A5G2QSE8	Keratin 3	1.48	-0.75

KRT75	F1SGI7	Keratin 75	1.37	-1.05
KRTCAP2	A0A287AU94	Keratinocyte-associated protein 2	1.80	-0.78
LAMTOR5	Q66X52	Ragulator complex protein LAMTOR5	2.00	1.26
LAS1L	A0A8W4FG52	LAS1 like ribosome biosis factor	1.90	0.38
LASP1	A0A287A8S0	LIM and SH3 protein 1	1.33	-0.42
LCN2	A0A287BJ65	Lipocalin 2	2.39	2.51
LCP1	F1RK02	Lymphocyte cytosolic protein 1	1.71	0.44
LEPROT	B9TRX0	Leptin receptor gene-related protein	1.74	1.03
LGALS1	Q49I35	Galectin-1	1.67	-0.52
LIMD1	F1SNZ3	LIM domain containing 1	2.43	-0.83
LIMS1	A0A5G2QPY8	LIM zinc-binding domain-containing protein	1.61	0.50
LIPG	I3LUA0	Lipoprotein lipase	1.40	1.41
LOC1005	I3LS74	Antileukoproteinase	2.61	2.74
LOC1005	A0A287BBT3	Nuclear autoantigen Sp-100	1.43	0.66
LOC1005	F1RV94	Transmembrane protein 14C	1.67	-2.80
LOC1005	F1RL06	Ig-like domain-containing protein	1.50	-1.53
LOC1007	F1SNU4	15 kDa protein B-like	2.00	2.78
LOC1021	A0A287AU92	Dehydrogenase/reductase SDR family member on cl	1.76	-0.68
LOC1065	A0A286ZYZ5	Serpin domain-containing protein	2.36	-2.11
LOC1102	A0A286ZP11	Protein S100	1.65	1.38
LOC1102	F1RGX4	Globin family profile domain-containing protein	2.33	-1.62
LOC1102	A0A5G2QTP8	Ig-like domain-containing protein	1.99	2.56
LOC1102	A0A287B0T7	Chromobox protein homolog 3 isoform X1	1.76	0.55
LPP	I3LUY1	LIM domain containing preferred translocation partn	1.82	-0.73
LRRC8C	F1S4E4	Leucine rich repeat containing 8 VRAC subunit C	1.94	0.53
LRRK2	F1SHN7	non-specific serine/threonine protein kinase	2.23	0.45
LTBP2	A0A5G2R7P3	Latent transforming growth factor beta binding prote	2.18	1.08
LTBP4	A0A8W4FF41	Latent transforming growth factor beta binding prote	2.06	0.61
LTF	Q6YT39	Lactotransferrin	2.27	2.51
LUC7L2	A0A5G2QA69	LUC7 like 2, pre-mRNA splicing factor	2.20	0.57
LUM	F1SQ09	Lumican	1.34	-0.91
LUZP1	I3LNF8	Leucine zipper protein 1	2.30	-0.79
LYN	F1RT53	Tyrosine-protein kinase	2.03	0.44
LYPLA2	I3LHP2	palmitoyl-protein hydrolase	2.10	0.56
LYVE1	I3LTD0	Lymphatic vessel endothelial hyaluronan receptor 1	1.54	-0.53
LZIC	A0A287A837	Leucine zipper and CTNNBIP1 domain containing	1.71	0.83
LZTFL1	F1SNZ1	Leucine zipper transcription factor-like protein 1	1.36	-0.38
MACO1	Q2TLZ2	Macoilin	1.32	-0.96
MAP1S	F1S939	Microtubule associated protein 1S	1.68	0.42
MAP2K5	A0A5G2R9B1	Mitogen-activated protein kinase kinase 5	1.87	-1.10
MAP4	A0A286ZXD9	Microtubule-associated protein	1.99	-0.83
MAPK1	A0A5G2RG05	Mitogen-activated protein kinase	1.44	0.39
MAPKAPK	A0A5K1UJP6	non-specific serine/threonine protein kinase	1.59	0.49
MARCKS	A0A287BRL8	Myristoylated alanine rich protein kinase C substrate	2.43	-0.80
MARCKSL	A0A287B4N8	MARCKS like 1	2.46	-0.91
MATR3	F1RGI7	Matrin 3	1.66	-0.40
MCFD2	K7GQY4	Multiple coagulation factor deficiency 2, ER cargo re	2.14	1.48
MDC1	Q767L8	Mediator of DNA damage checkpoint protein 1	2.00	0.71
MED16	F1S6T0	Mediator of RNA polymerase II transcription subunit	1.52	0.92
METTL9	I3LID3	Methyltransferase like 9	1.43	0.54

MICAL3	A0A287B5Y3	F-actin monooxygenase	1.61	-1.33
MIDEAS	F1S3I8	Mitotic deacetylase associated SANT domain proteir	2.00	0.84
MMP8	F1SV69	Matrix metallopeptidase 8	1.44	1.57
MMP9	A0A287AZD5	Matrix metallopeptidase 9	2.32	2.21
MOBKL3	F2Z5T8	MOB-like protein phocein	1.54	0.41
MPC1	A0A5G2R834	Mitochondrial pyruvate carrier	1.49	-0.49
MPC2	A0A5G2REE1	Mitochondrial pyruvate carrier	1.80	-0.41
MPEG1	A0A8W4F8T8	Macrophage-expressed gene 1 protein	1.38	-0.85
MPO	K7GRV6	Myeloperoxidase	2.36	2.54
MRC1	A0A5G2R0H2	Mannose receptor C-type 1	1.50	-0.38
MRPS17	F1RIU0	28S ribosomal protein S17, mitochondrial	1.45	-1.26
MRPS25	A0A0M3KL55	Interferon alpha-inducible protein 6	1.38	-0.61
MSRA	F1RJP8	peptide-methionine (S)-S-oxide reductase	1.52	0.61
MT-ATP8	Q35914	ATP synthase protein 8	2.41	1.27
MT-CO2	Q69GF7	Cytochrome c oxidase subunit 2	2.43	-0.91
MTHFR	A0A287B634	Methylenetetrahydrofolate reductase	1.80	-0.53
MTM1	A0A286ZR95	Phosphatidylinositol-3,5-bisphosphate 3-phosphata	1.65	0.78
MT-ND3	O79880	NADH-ubiquinone oxidoreductase chain 3	2.15	0.99
MT-ND5	Q9TDR1	NADH-ubiquinone oxidoreductase chain 5	1.42	-0.54
MVB12A	A0A287AP77	Multivesicular body subunit 12A	1.57	-0.62
MYADM	A0A5G2QYN7	Myeloid associated differentiation marker	2.38	-0.77
MYDGF	M3UZ63	Myeloid derived growth factor	1.94	-0.38
MYG1	F1SFR1	MYG1 exonuclease	1.31	1.13
MYL6	P60662	Myosin light polypeptide 6	2.27	1.02
MYO1F	A0A5G2R1L5	Myosin IF	1.70	0.78
MYO1G	A0A287A8I5	Myosin IG	1.67	0.42
MYZAP	F1RZH1	Myocardial zonula adherens protein	1.75	0.44
NAPA	A0A5G2QNT7	NSF attachment protein alpha	2.41	0.47
NASP	A0A5G2QJ28	Nuclear autoantigenic sperm protein	1.41	0.37
NAT10	F1SGS7	RNA cytidine acetyltransferase	1.69	0.44
NCALD	F2Z561	Neurocalcin delta	1.78	1.03
NCBP1	A0A5G2QD06	Nuclear cap binding protein subunit 1	2.23	0.46
NCF1	F1RJL1	Neutrophil cytosolic factor 1	1.80	1.02
NCF4	A0A5G2R098	Neutrophil cytosolic factor 4	1.87	0.64
NCKAP1L	K9IWB5	NCK associated protein 1 like	1.94	0.50
NCOA2	F1RU09	Nuclear receptor coactivator	2.61	-0.89
NDOR1	A0A481CV86	NADPH-dependent diflavin oxidoreductase 1	1.60	0.73
NDUFB4	A0A287BPW8	NADH dehydrogenase [ubiquinone] 1 beta subcomp	1.89	-0.60
NDUFB7	F1SCH1	NADH dehydrogenase [ubiquinone] 1 beta subcomp	1.75	1.03
NDUFC2	A0A480JRW3	NADH dehydrogenase [ubiquinone] 1 subunit C2	2.26	-1.00
NDUFS8	A0A286ZUN9	NADH dehydrogenase [ubiquinone] iron-sulfur prote	2.19	-0.73
NEDD4L	A0A5G2R0L1	HECT-type E3 ubiquitin transferase	1.41	-0.58
NELFE	F1RQW5	Negative elongation factor E	1.83	-0.78
NF2	A0A480KT19	Merlin isoform 1	1.58	0.74
NOSIP	F1RHW0	Nitric oxide synthase-interacting protein	1.67	-0.53
NOTCH3	I3LB93	Notch receptor 3	1.95	-0.84
NPC2	O97763	NPC intracellular cholesterol transporter 2	1.55	-0.52
NPG1	A0A5G2QNY2	Protegrin-1	2.17	1.65
NQO1	A0A5G2QPB5	NAD(P)H quinone dehydrogenase 1	1.33	-0.43
NR3C1	A0A287B660	Glucocorticoid receptor	2.39	-0.65

NT5C2	A0A287BBC4	5'-nucleotidase, cytosolic II	1.42	0.56
NT5C3A	I3LCJ3	5'-nucleotidase	1.76	0.66
NT5DC1	K7GPB8	5'-nucleotidase domain containing 1	1.45	0.52
NUP153	A0A481AFG9	Nuclear pore complex protein Nup153 isoform X1	1.69	-0.48
NUP35	A0A480QBL1	Nucleoporin NUP53	1.82	-0.91
NUP62	A0A287AZ57	Nucleoporin 62	1.44	0.56
OGN	A0A0H5ANC0	Osteoglycin	1.78	-0.85
OLFML1	A0A286ZY90	Olfactomedin-like domain-containing protein	1.90	-0.95
OLFML2A	A2BD09	Olfactomedin-like protein 2A	1.44	-0.85
OLFML3	H6UWK6	Olfactomedin like 3	1.64	-0.56
OLR1	Q9TTK7	Oxidized low-density lipoprotein receptor 1	2.36	2.78
OSBPL2	M3V843	Oxysterol-binding protein	2.61	0.66
OSTF1	Q8MJ49	Osteoclast-stimulating factor 1	2.19	0.71
OTULINL	A0A287AVS5	Inactive ubiquitin thioesterase FAM105A	2.33	0.87
PADI4	F1SUR0	Protein-arginine deiminase	2.61	2.77
PALLD	A0A287BPI5	Palladin isoform X4	1.38	-0.35
PALM	A0A286ZKH7	Paralemmin	1.83	-0.40
PALS1	F1SA41	Protein PALS1	2.05	0.56
PAM	A0A287A5G2	Peptidylglycine alpha-amidating monooxygenase	1.45	1.29
PAPOLA	A0A287BSI1	Poly(A) polymerase	1.53	0.47
PARK7	Q0R678	protein deglycase	1.84	-0.54
PARVG	F1SJU3	Parvin gamma	1.76	0.66
PAWR	A0A287ARB3	Pro-apoptotic WT1 regulator	2.41	-0.86
PCBP1	A0A8W4FAT6	Poly(rC) binding protein 1	2.48	-0.55
PCOLCE	I3LEE6	Procollagen C-endopeptidase enhancer	1.90	-0.71
PDLIM1	F1SC51	PDZ and LIM domain 1	2.08	-0.54
PDLIM2	F1RMA7	PDZ and LIM domain 2	2.36	-1.35
PDLIM3	Q6QGC0	PDZ and LIM domain protein 3	1.42	-0.89
PDLIM4	F1RJV5	PDZ and LIM domain 4	1.39	-0.79
PDS5B	A0A286ZSB7	PDS5 cohesin associated factor B	2.27	0.39
PEA15	A0A287AC69	Astrocytic phosphoprotein PEA-15	1.59	1.45
PEF1	A0A5G2R489	Penta-EF-hand domain containing 1	1.50	0.40
PFKFB2	A0A287A3X9	6-phosphofructo-2-kinase/fructose-2,6-biphosphat	1.31	0.57
PFN2	I3L660	Profilin	2.19	-0.73
PGLS	A0A286ZS31	6-phosphogluconolactonase	2.33	0.55
PGLYRP1	A0A286ZI97	Peptidoglycan-recognition protein	2.34	1.99
PGM5	F1SJE6	Phosphoglucomutase 5	2.30	-0.57
PGRMC1	Q95250	Membrane-associated progesterone receptor comp	1.39	0.59
PHGDH	A5GFY8	D-3-phosphoglycerate dehydrogenase	2.38	0.93
PHIP	A0A5G2QXE1	Pleckstrin homology domain interacting protein	2.14	1.33
PIGS	I3LLF3	GPI transamidase component PIG-S	1.34	0.36
PLD3	A0A287AQV3	Phospholipase D family member 3	2.35	-0.56
PLEK	A0A5G2QT56	Pleckstrin	2.22	0.86
PLEKHA2	A0A287BNI0	Pleckstrin homology domain containing A2	1.33	-0.36
PLEKHA6	A0A5G2RE29	Pleckstrin homology domain containing A6	1.65	-0.87
PLPP6	F1SK43	Phospholipid phosphatase 6	1.62	-0.82
PLS1	A0A287BI79	Plastin 1	1.80	0.49
PLSCR1	A0A5G2QX76	Phospholipid scramblase	1.72	-0.82
PMAP37	P49932	Antibacterial peptide PMAP-37	2.38	2.49
PMF1	F1RLQ4	Polyamine modulated factor 1.SPMF1	1.43	0.72

PML	A0A287AZS0	PML nuclear body scaffold	1.46	0.48
POLDIP2	I3LMX3	DNA polymerase delta interacting protein 2	1.35	-0.37
PON2	F1SFA2	Paraoxonase	1.69	0.53
POSTN	F1RS37	Periostin	3.26	-1.11
PPHLN1	A0A287AKZ6	Periphilin 1	2.15	-1.37
PPIA	P62936	Peptidyl-prolyl cis-trans isomerase A	1.64	-0.42
PPIF	F1S2E3	Peptidyl-prolyl cis-trans isomerase	1.45	1.04
PPIL1	A0A287AFF5	Peptidyl-prolyl cis-trans isomerase	1.94	-0.53
PPM1F	F1RL04	Protein phosphatase, Mg2+/Mn2+ dependent 1F	2.21	0.49
PPP1R11	B6ICU5	E3 ubiquitin-protein ligase PPP1R11	2.04	-1.02
PPP1R12/	A0A5G2R8E1	Protein phosphatase 1 regulatory subunit	1.99	-0.38
PPP2R1B	P54613	Serine/threonine-protein phosphatase regulatory su	1.61	0.74
PPP2R5E	A0A287B3M4	Serine/threonine-protein phosphatase 2A 56 kDa reg	2.61	0.53
PPP3R1	A0A5G2Q9D7	Protein phosphatase 3 regulatory subunit B, alpha	1.81	1.05
PR39	P80054	Antibacterial protein PR-39	2.39	2.08
PRDX1	A0A286ZND5	Peroxiredoxin-1	1.80	-0.52
PRDX2	A0A287A690	Peroxiredoxin 2	1.88	-0.76
PRELP	F1S6B4	Prolargin	1.79	-0.84
PREX1	A0A286ZUE0	Phosphatidylinositol-3,4,5-trisphosphate dependen	2.11	0.63
PRKCB	K9J4L8	Protein kinase C	1.60	0.55
PRPF38A	I3LP32	Pre-mRNA-splicing factor 38A	1.60	0.38
PRRC2C	A0A286ZS85	Proline rich coiled-coil 2C	2.00	-0.64
PRTN3	F1S6S9	Proteinase 3	2.43	3.03
PSMB3	A0A286ZN52	Proteasome subunit beta	2.18	-0.39
PSME3	P61291	Proteasome activator complex subunit 3	1.60	0.45
PTGES2	F1RRY6	Prostaglandin E synthase 2	1.51	0.42
PTI	F1SD24	BPTI/Kunitz inhibitor domain-containing protein	1.93	1.88
PTPN6	A0A480TLF2	Tyrosine-protein phosphatase non-receptor type	1.39	0.41
PTPN9	F1SJ71	Protein tyrosine phosphatase non-receptor type 9	1.39	0.38
PTPRE	A0A5G2R524	Protein tyrosine phosphatase receptor type E	1.46	0.46
PYM1	A0A287B9S4	PYM homolog 1, exon junction complex associated f	1.65	-0.66
QDPR	Q8MJ30	Dihydropteridine reductase	1.80	-0.41
QTRT1	F1S586	Queuine tRNA-ribosyltransferase catalytic subunit 1	1.32	-0.49
RAB31	A0A480QQS9	RAB31, member RAS onco family	1.56	0.76
RAB3D	A0A287BLN4	Ras-related protein Rab-3	2.29	1.33
RAC2	A0A5G2R1K1	Rac family small GTPase 2	1.68	0.89
RAD50	A0A287BNG5	RAD50 double strand break repair protein	1.65	0.35
RALY	A0A286ZLQ8	RALY heterogous nuclear ribonucleoprotein	1.83	-0.53
RAMP2	F6PWN2	Receptor activity modifying protein 2	1.57	1.22
RASGRP2	F1RQP7	RAS guanyl releasing protein 2	2.39	1.02
RASSF2	A0A287B3Z2	Ras association domain family member 2	1.37	0.42
RASSF8	F1SG73	Ras association domain family member 8	1.42	-0.89
RBBP7	A0A5G2RBI2	RB binding protein 7, chromatin remodeling factor	1.94	0.71
RBM14	A0A287B401	RNA binding motif protein 14	2.46	-0.47
RBM39	A0A287BAQ0	RNA binding motif protein 39	2.13	0.37
RBMXL1	K7GNV0	RBMX like 1	1.67	-0.57
RCN3	I3LMU6	Reticulocalbin 3	1.42	0.37
REPS1	F1S6Z2	RALBP1 associated Eps domain containing 1	1.62	-0.41
RETN	Q6QR67	Resistin	2.11	1.55
RHAG	F1RPZ7	Rh associated glycoprotein	2.32	-1.19

RNASET2	A0A287ALP1	Ribonuclease T2	2.18	0.65
RNF123	A0A286ZKD3	Ring finger protein 123	1.89	-0.67
RNH1	A0A5S6IIV1	Ribonuclease inhibitor	2.45	0.78
RPE	A0A286ZQY9	Ribulose-phosphate 3-epimerase	2.33	0.85
RPL14	A1XQU3	60S ribosomal protein L14	1.75	-0.39
RPL21	P49666	60S ribosomal protein L21	2.19	-0.53
RPL23	P62831	60S ribosomal protein L23	1.59	-0.45
RPL27	A1XQU5	60S ribosomal protein L27	1.93	-0.65
RPL28	A0A287AJT7	60S ribosomal protein L28	1.99	-0.48
RPL29	Q95281	60S ribosomal protein L29	1.93	-0.82
RPL32	Q6QAT0	60S ribosomal protein L32	1.72	-0.35
RPL35	Q29361	60S ribosomal protein L35	1.37	-0.91
RPL35A	A0A286ZLH8	60S ribosomal protein L35a	2.39	-1.12
RPL7	A0A8W4FHN2	Ribosomal protein L7	1.99	-0.45
RPRD1B	F2Z5H3	Regulation of nuclear pre-mRNA domain-containing	1.74	0.46
RPS16	Q29201	40S ribosomal protein S16	2.13	-0.62
RPS25	F2Z5G8	40S ribosomal protein S25	1.49	-0.51
RPS27A	A0A287AZA7	Ubiquitin-40S ribosomal protein S27a	3.95	-0.85
RPS3A	F2Z5C7	40S ribosomal protein S3a	1.67	-0.40
RPS6KB1	A0A481C7T7	Ribosomal protein S6 kinase	1.94	-0.70
RSC1A1	A0A5G2QHK1	Regulator of solute carriers 1	2.55	-0.77
RTKN	A0A287BF14	Rhotekin	1.36	-0.58
S100A1	K7GQ50	Protein S100-A1	1.64	1.95
S100A11	P31950	Protein S100-A11	1.77	1.28
S100A12	P80310	Protein S100-A12	3.20	3.44
S100A13	A0A287BEK8	S100 calcium binding protein A13	2.00	1.02
S100A8	C3S7K5	Protein S100	2.29	2.60
S100A9	K7GME6	S100 calcium binding protein A9	2.04	2.30
S1PR1	I3LIS0	Sphingosine 1-phosphate receptor 1	1.31	0.64
SASH3	A0A480KK25	SAM and SH3 domain containing 3	1.47	0.62
SBSPON	A0A480WBM1	Somatomedin B and thrombospondin type 1 domain	2.00	-0.88
SCAMP3	F1RLJ4	Secretory carrier-associated membrane protein	1.43	-0.38
SCGB1A1	F1RPX3	Uteroglobin	1.56	1.92
SDS	K9IVJ5	L-serine ammonia-lyase	1.68	2.18
SEC14L1	F1RZ70	SEC14 like lipid binding 1	1.63	0.82
SELENOF	A1Z623	Selenoprotein F	1.34	0.49
SELENOI	I3L5E9	Selenoprotein I	1.59	-0.93
SELENOM	A0A8W4FJJ8	Selenoprotein M	1.52	0.75
SEMA4A	A0A287AME3	Semaphorin 4A	2.02	1.45
SEMA4C	F1STD1	Semaphorin 4C	1.62	-0.82
SEMA7A	A0A8W4FHQ7	Semaphorin 7A (John Milton Hagen blood group)	1.44	1.00
SEPHS1	A0A286ZVF7	selenide, water dikinase	1.78	0.38
SERPINB1	F2Z5B1	Serpin family B member 1	1.49	1.05
SERPINB1	F1SMW8	Serpin family B member 10	1.60	1.09
SF3A1	A0A287ACP7	Splicing factor 3a subunit 1	2.17	-0.43
SF3A3	F1SV40	Splicing factor 3A subunit 3 isoform 1	1.82	0.35
SFTPC	Q3MSM1	Pulmonary surfactant-associated protein C	3.26	-1.66
SFTPD	Q9N1X4	Pulmonary surfactant-associated protein D	1.57	-0.76
SFXN3	A0A287AHF5	Sidoreflexin	1.69	-0.35
SGTA	A0A5G2QXK3	Small glutamine rich tetratricopeptide repeat co-cha	2.36	0.65

SH3BP1	A0A8W4FNZ4	SH3 domain binding protein 1	1.56	0.45
SH3GL1	F1S7L8	SH3 domain containing GRB2 like 1, endophilin A2	2.39	-0.58
SH3PXD2I	A0A5G2QR03	SH3 and PX domains 2B	1.64	-0.46
SHMT2	A0A287BKR2	Serine hydroxymethyltransferase	2.00	0.47
SHROOM:	K7GQZ0	Shroom family member 2	2.33	-0.98
SHTN1	F1S4S1	Shootin-1	1.40	-0.41
SIPA1	F1RRK2	Signal-induced proliferation-associated 1	1.57	0.36
SKAP2	A0A480KBE3	Src kinase associated phosphoprotein 2	1.40	0.50
SKP1	A0A287ARU4	S-phase kinase-associated protein 1	1.63	0.58
SLC12A2	A0A480HW11	Solute carrier family 12 member 2	1.44	-0.37
SLC25A2(	F1SKK1	Mitochondrial carnitine/acylcarnitine carrier protein	1.62	0.58
SLC2A3	O62787	Solute carrier family 2, facilitated glucose transporte	1.43	0.98
SLC34A2	F1S5A6	Sodium-dependent phosphate transport protein 2B i	2.70	-0.93
SLC44A2	F1S584	Choline transporter-like protein 2	2.10	0.59
SLC4A1	K7GR72	Anion exchange protein	2.61	-1.68
SLC66A3	F1SAA6	Solute carrier family 66 member 3	1.85	-0.57
SLIT2	F1S5C4	Slit guidance ligand 2	1.48	-1.07
SMTN	F1RPD7	Smoothelin	2.90	-0.94
SNAP29	F1RKY1	Synaptosome associated protein 29	1.94	-0.63
SNCG	A7YX24	Gamma-synuclein	1.47	-1.31
SNRPE	I3LDW9	Small nuclear ribonucleoprotein E	2.13	-0.63
SNRPN	I3LBT8	Small nuclear ribonucleoprotein-associated protein	1.82	-0.48
SNTB2	I3LDU9	Syntrophin beta 2	1.61	-0.38
SNW1	I3L897	SNW domain-containing protein 1	1.39	-0.50
SORBS2	F1RZN9	Sorbin and SH3 domain containing 2	1.99	-0.93
SORBS3	F1RMA6	Sorbin and SH3 domain containing 3	1.60	-0.50
sp|P0076	P00761	Trypsin	1.62	-0.73
sp|P0184	P01846	Ig lambda chain C region	2.19	-0.98
sp|Q69DI	Q69DK8	Complement C1s subcomponent	1.65	-0.65
SPARC	P20112	SPARC	1.64	-0.40
SPTA1	A0A5G2R924	Spectrin alpha, erythrocytic 1	2.33	-0.95
SPTB	A0A287A113	Spectrin beta chain	2.43	-1.04
SPTBN2	A0A287B3A4	Spectrin beta chain	1.64	0.71
SQSTM1	A0A287BLE1	Sequestosome 1	1.49	0.58
SRPK2	A0A287A5X3	SRSF protein kinase 2	2.23	0.93
SRPX	K7GKF8	Sushi repeat containing protein X-linked	1.36	-0.98
SRSF10	A0A287B770	Serine and arginine rich splicing factor 10	1.96	0.63
SSB	F1S1V1	Lupus La protein	2.41	0.46
ST14	A0A287AP26	ST14 transmembrane serine protease matriptase	2.29	1.32
STK24	A0A287B0C4	Serine/threonine kinase 24	2.46	0.50
STK38L	I3LN61	Serine/threonine kinase 38 like	1.69	0.58
STX11	F1S731	Syntaxin 11	1.61	0.42
STX17	A0A481CAI8	Syntaxin 17	1.33	0.90
STX3	A0A8W4FDM0	Syntaxin 3	1.99	1.13
STXBP2	A0A5G2QA44	Syntaxin binding protein 2	2.39	0.64
SULT1C4	I3LC84	Sulfotransferase	1.53	0.55
SUSD2	A0A8W4FGK0	Sushi domain containing 2	2.04	0.50
SWAP70	A0A5G2QQG7	Switching B cell complex subunit SWAP70	2.39	0.45
SYAP1	A0A287AX76	Synapse-associated protein 1	2.39	-1.00
SYMPK	F1RM40	Symplekin scaffold protein	1.83	0.49

SYNPO2	A0A287AUH9	Synaptopodin 2	2.02	-0.95
TACC1	A0A287BJ18	Transforming acidic coiled-coil-containing protein 1	1.64	-0.41
TACSTD1	Q75QW1	Epithelial cell adhesion molecule	1.39	0.41
TAGLN	A0A287AZK2	Transgelin	1.40	-0.50
TAGLN2	F1RJ93	Transgelin	2.20	-0.53
TAOK2	A0A287ATS3	Serine/threonine-protein kinase TAO2 isoform X1	1.61	-0.97
TAPT1	I3L7J1	Transmembrane anterior posterior transformation 1	1.36	-0.43
TARS1	F1SP18	threonine--tRNA ligase	2.52	0.45
TBC1D5	A0A5G2QJK6	TBC1 domain family member 5	1.69	-0.55
TBCB	Q8HXL4	Cytoskeleton-associated protein 1	1.67	-0.39
TCN1	A0A287A0W9	Transcobalamin-1	2.36	2.93
TF	P09571	Serotransferrin	1.83	-0.96
TFAM	Q5D144	Transcription factor A, mitochondrial	1.81	-0.45
TFG	I3LNI2	Trafficking from ER to golgi regulator	1.39	-0.36
TFIIH	Q767M2	General transcription factor IIH subunit 4	1.38	0.56
THAP11	F1S2K4	THAP domain containing 11	1.33	-0.43
THOC2	A0A287AC85	THO complex subunit 2	2.07	0.42
TIA1	I3LRX5	TIA1 cytotoxic granule associated RNA binding protei	1.68	-0.68
TINAGL1	F1SVA2	Tubulointerstitial nephritis antigen like 1	2.05	-1.11
TKFC	F1RKQ4	Triokinase/FMN cyclase	1.52	0.39
TM7SF3	F1SG79	Transmembrane 7 superfamily member 3	1.44	1.04
TMBIM1	A0A5G2RAH9	Transmembrane BAX inhibitor motif containing 1	1.40	-0.86
TMED1	A0A287BCX9	Transmembrane p24 trafficking protein 1	1.75	-1.29
TMED10	A0A286ZV95	Transmembrane emp24 domain-containing protein :	1.96	-0.38
TMEM106	A0A287B4J0	Transmembrane protein 106B	1.34	-0.35
TMEM109	A0A5G2RAI4	Transmembrane protein 109	1.44	-0.76
TMEM126	I3LJK4	Transmembrane protein 126A	2.36	1.33
TMEM263	A0A5G2QVT2	Transmembrane protein 263	1.54	-0.52
TMOD3	A0A5G2R425	Tropomodulin 3	1.91	0.52
TMX3	A0A5G2R124	Protein disulfide-isomerase TMX3 isoform 1	1.62	0.43
TMX4	I3LB23	Thioredoxin related transmembrane protein 4	1.34	-0.45
TNC	Q29116	Tenascin	1.49	0.85
TNFAIP2	A0A5G2RNN3	TNF alpha induced protein 2	1.44	0.48
TNFAIP8L:	F1SSY0	TNF alpha induced protein 8 like 2	2.00	1.03
TNPO2	F1SEX5	Transportin 2	1.58	0.45
TNPO3	A0A287BR50	Transportin 3	1.95	0.54
TNS1	A0A5G2R986	Tensin 1	2.04	-0.56
TOM1L2	A0A287BAN7	TOM1-like protein 2 isoform 3	1.61	-0.48
TOP1	I3LHM3	DNA topoisomerase I	1.79	0.43
tr|A0A07!	A0A075B7H9	Ig-like domain-containing protein	1.53	-0.99
tr|A0A07!	A0A075B7I9	Ig-like domain-containing protein	1.62	-1.15
tr|A0A28(	A0A286ZRF3	Serpin domain-containing protein	2.39	-0.56
tr|A0A28	A0A287AAG8	VWFA domain-containing protein	2.11	1.28
tr|A0A28	A0A287ACY9	Nuclear envelope pore membrane protein POM 121(	1.56	-1.19
tr|A0A28	A0A287AEW5	C-type lectin domain-containing protein	2.26	-2.13
tr|A0A28	A0A287ANQ8	C-type lectin domain-containing protein	1.37	-1.67
tr|A0A28	A0A287ANY2	Ig-like domain-containing protein	1.90	0.69
tr|A0A28	A0A287APR1	60S ribosomal protein L18a	1.83	-0.40
tr|A0A28	A0A287B046	Uncharacterized protein	1.61	1.03
tr|A0A28	A0A287BDI2	Peptidase S1 domain-containing protein	1.80	1.83

tr|A0A287	A0A287BIA5	Uncharacterized protein	3.26	-1.19
tr|A0A287	A0A287BN06	Pregnancy zone protein	1.35	-1.16
tr|A0A480	A0A480RQR3	arylamine N-acetyltransferase	1.35	0.44
tr|A0A5G2	A0A5G2QJS5	Acidic leucine-rich nuclear phosphoprotein 32 family	2.58	0.83
tr|A0A5G2	A0A5G2QN35	UEV domain-containing protein	1.67	-0.55
tr|A0A5G2	A0A5G2QNQ5	Nesprin-1	1.64	-0.42
tr|A0A5G2	A0A5G2QRW3	Globin family profile domain-containing protein	2.25	-1.58
tr|A0A5G2	A0A5G2R996	KASH domain-containing protein	1.72	0.38
tr|A0A8W	A0A8W4F8U6	Ig-like domain-containing protein	1.48	-1.40
tr|A0A8W	A0A8W4FC15	Ig-like domain-containing protein	2.33	-1.78
tr|A0A8W	A0A8W4FGJ9	QLQ domain-containing protein	1.94	-0.79
tr|A0A8W	A0A8W4FJA1	Ig-like domain-containing protein	1.37	-1.05
tr|A0A8W	A0A8W4FN52	Ubiquitin-like protein 5	1.97	0.55
tr|A0A8X9	A0A8X9AEV6	Cytochrome P450 2J2	1.47	0.67
tr|F1RYZ0	F1RYZ0	60S acidic ribosomal protein P2	1.68	0.53
tr|F1S9A4	F1S9A4	EF-hand domain-containing protein	2.13	1.22
tr|F1SD56	F1SD56	A-kinase anchor protein 2 C-terminal domain-contai	1.50	-0.89
tr|F1SDT0	F1SDT0	Junctophilin-2	1.65	-0.54
tr|F1ST28	F1ST28	Macrosialin isoform A	1.50	0.71
tr|F1STC5	F1STC5	Ig-like domain-containing protein	1.37	-0.93
tr|I3LEF9	I3LEF9	C-type lectin domain-containing protein	1.56	-1.87
tr|I3LLX8	I3LLX8	Sulfotransferase	2.08	0.70
tr|I3LSD3	I3LSD3	60S ribosomal protein L13	1.68	-0.49
TRA2B	Q06AA7	TRA2B	2.43	0.74
TRAPPC1	F1RT12	Trafficking protein particle complex subunit 11	2.13	0.48
TRAPPC1	A0A287A5W6	Trafficking protein particle complex subunit 13	1.56	0.74
TRAPPC3	A0A287AWQ9	Trafficking protein particle complex subunit	1.49	0.37
TRAPPC4	A0A286ZKY8	Trafficking protein particle complex subunit 4	1.32	0.36
TRIP6	I3LCI1	Thyroid hormone receptor interactor 6	2.22	-0.73
TRIQK	A0A287AL49	Triple QxxK/R motif-containing protein	2.06	-1.05
TRMT11	A0A481AJX3	tRNA (guanine(10)-N2)-methyltransferase homolog	1.40	-0.92
TRPM7	F1RYM3	non-specific serine/threonine protein kinase	1.61	-1.56
TSC22D4	F1RMY6	TSC22 domain family member 4	1.67	-0.97
TSPAN14	A0A287AA08	Tetraspanin 14	2.17	0.92
TUBB2A	A0A286ZN27	Tubulin beta chain	1.45	-0.57
TUBGCP2	A0A480VJI0	Gamma-tubulin complex component	2.43	0.44
TWF2	A0A5G2Q7I8	Twinfilin actin binding protein 2	1.87	0.36
TXNDC12	A0A481A893	Thioredoxin domain-containing protein 12	1.62	0.51
UBAP1	F1SEA8	Ubiquitin associated protein 1	1.44	-0.75
UBASH3B	A0A287BBF2	Ubiquitin associated and SH3 domain containing B	1.59	0.58
UBE2G2	I3LJ21	Ubiquitin conjugating enzyme E2 G2	1.71	0.64
UBE2H	A0A287AB80	Ubiquitin conjugating enzyme E2 H	1.63	0.51
UBE2Z	I3LSU8	Ubiquitin-conjugating enzyme E2 Z	1.65	0.36
UCHL3	A0A5G2QNC6	Ubiquitin carboxyl-terminal hydrolase	1.42	0.64
UCHL5	Q06AT3	Ubiquitin carboxyl-terminal hydrolase isozyme L5	2.43	0.50
UFSP2	F1RZQ3	UFM1 specific peptidase 2	1.91	0.55
UGP2	A0A481C881	UTP--glucose-1-phosphate uridylyltransferase	1.89	0.46
UPF3B	F1RUA3	UPF3B regulator of nonsense mediated mRNA decay	2.00	-1.00
UROD	F1S365	Uroporphyrinogen decarboxylase	1.98	-0.54
USE1	F1S960	Vesicle transport protein USE1	1.46	-0.43

VAPB	A0A287B186	VAMP associated protein B and C	2.08	-0.38
VAV1	A0A286ZQX2	Proto-oncogene vav	2.05	0.48
VCAN	F1REZ2	Versican	1.47	-0.54
VIM	P02543	Vimentin	2.13	-0.66
VNN2	F1S3Q9	Vanin 2	1.34	1.57
VSIR	A0A8W4FCG3	V-set immunoregulatory receptor	1.35	0.79
VTA1	A0A5G2QAP4	Vesicle trafficking 1	1.60	0.38
WASL	F1SMK2	WASP like actin nucleation promoting factor	1.75	-0.64
WDR45B	D7RA17	WD repeat domain 45B	1.60	0.45
WDR70	F1SN94	WD repeat domain 70	1.97	-0.54
WDR77	F1S8D4	WD repeat domain 77	2.19	-0.39
WDR81	M3VHA7	WD repeat domain 81	1.72	-0.43
WRAP53	F1ST42	WD repeat containing antisense to TP53	1.49	0.56
XPO6	F1RFG5	Exportin-6	1.57	0.58
YAP1	A0A286ZNK4	Yes1 associated transcriptional regulator	2.74	-0.73
YBX1	A0A8W4FPA2	Y-box binding protein 1	2.23	-0.83
ZC3H11A	F1S6B2	Zinc finger CCCH domain-containing protein 11A iso	1.74	-0.70
ZC3H14	A0A287BD48	Zinc finger CCCH domain-containing protein 14	2.23	-0.81
ZFYVE16	F1RF24	Zinc finger FYVE domain-containing protein 16	1.55	-0.39
ZMPSTE24	I3LR30	CAAX prenyl protease	2.36	-0.61
ZMYND11	A0A480U735	Zinc finger MYND domain-containing protein 11 isofo	1.38	0.55
ZNF143	A0A5G2QTH5	Zinc finger protein 143	1.71	-0.68
ZNF207	A0A287AA44	Zinc finger protein 207	1.80	-0.67
ZNHIT2	F1RQU8	Zinc finger HIT-type containing 2	1.32	0.78
ZYX	F1SRU2	Zyxin	2.23	-1.16

Shows all significantly differentially expressed proteins identified per group. Columns in order as follows: Gene names from uniprot, Uniprot ID, Protein name, negative log10 q-value (FDR corrected p-value) as well as effect size.
