# Supplementary material for: Unraveling Molecular and Functional Responses Across 3 Lung Injury Models to Expand the Donor Lung Pool
Source: Transplantation. 2025 Feb 19;109(7):1166–74. doi: 10.1097/TP.0000000000005353 (PMC12180699; doi:10.1097/TP.0000000000005353)
Supplement: Supplementary file 6 [file tpa-109-1166-s006.pdf]

Table S5

Group	Uniquely upregulated pathways	Top 10 core enrichment proteins
LPS	Regulation of immune system process	LGALS13, TNFAIP3, LTF, THBS1, ALOX15, SPN, MTURN, IGFBP2, ORM1, GGT1
	Response to stress	S100A12, S100A8, MPO, AZU1, S100A9, OLR1, PGLYRP1, PR39, TNFAIP3, NPG1,
Gastric	T-cell proliferation	LGALS13, ANXA1, RAC2, SASH3, IGFBP2, STAT5A, IL18, AIF, RIPK3, PYCARD
VILI	Monocyte chemotaxis	CCR1, ANXA1, AIF1

Group	Uniquely downregulated pathways	Top 10 core enrichment proteins
LPS	Response to heat/temperature stimulus	MTOR, FGF1, CKM
Gastric	Complement activation	CFB, C2, CD46, C9, C7, C1QA, C4BPA, C8A, C6, C3, C8B, C1QC
	Cellular response to toxic substance/ detoxification	ADH5, SOD1, GSTM3, PARK7, HBZ, HBB
VILI	Lipid metabolic process	STAT5A, FDXR, ASAH1, DGKA, ACOX1, ABHD6, PDK3, MMUT, AOX1, PIP4K2A
	Phospholipid metabolic process	DPM1, INPPL1, ORMDL3, ACP6, GNPAT, NKX2-1, MTMR2, TAMM41, AGPAT1, MTM1
	Cellular lipid metabolic process	AUH, PTGES, HADH, ACSL5, DPM1, EPHX2, INPP5K, INPPL1, ORMDL3, ACP6
	Glycerophospholipid metabolic process	DGKA, ABHD6, PIP4K2A, DPM1, INPP5K, INPPL1, GNPAT, MTMR3, TAMM41, MTM1
	Immunoglobulin/B-cell mediated immune process	LOC100517145, C7, TGFB1, EXOSC3, C6, C3, C1QC, C5, FCER1G, C1QA, C4BPA
	Platelet activation	VWF, FGG, FCER1G

Shows uniquely upregulated and downregulated Gene Ontology Biological Process pathways per group, as well as top ten core enriched proteins per pathway. Proteins are named with their respective Gene name from uniprot.org.
