# Supplementary material for: Unraveling Molecular and Functional Responses Across 3 Lung Injury Models to Expand the Donor Lung Pool
Source: Transplantation. 2025 Feb 19;109(7):1166–74. doi: 10.1097/TP.0000000000005353 (PMC12180699; doi:10.1097/TP.0000000000005353)
Supplement: Supplementary file 7 [file tpa-109-1166-s007.pdf]

Table S6

LPS	Gene	Uniprot ID	Protein name	log10 q-val	Effect Size
	C3	P01025	Complement C3	55.62	16.07
	HP	Q8SPS7	Haptoglobin	54.81	15.91
	LOC106504547	A0A286ZYZ5	Serpin domain-containing protein	52.94	15.61
	GC	A0A287AHK1	Vitamin D-binding protein	42.20	13.93
	A1BG	A0A286ZUY9	Alpha-1-B glycoprotein	40.05	13.55
	A0A8W4FRI5_PIG	A0A8W4FRI5	Ig-like domain-containing protein	22.33	10.12
	TF	P09571	Serotransferrin	22.11	10.05
	LOC396685	F1SCD0	Serpin domain-containing protein	21.71	9.94
	SERPINC1	A0A480P2S5	Antithrombin-III	19.20	9.34
	PPP1CB	P61292	Serine/threonine-protein phosphatase PP1-beta catalytic subunit	10.33	6.84
	IGHM	A0A287ALC1	Immunoglobulin heavy constant mu	10.01	6.72
	AHSG	P29700	Alpha-2-HS-glycoprotein (Fragment)	7.87	5.95
	DSG4	I3L5R5	Desmoglein 4	6.35	-5.33
	A2M	A0A5G2Q8I9	Alpha-2-macroglobulin	4.86	4.65
	CALR	P28491	Calreticulin	4.24	4.34
	FLNA	A0A287AW81	Filamin A	3.72	4.05
	LOC100125542	A0A8W4FC14	Ig-like domain-containing protein	1.67	5.77
**VILI**	**Gene**	**Uniprot ID**	**Protein name**	**log10 q-val**	**Effect Size**
	ALB	P08835	Albumin	11.57	7.19
	TF	P09571	Serotransferrin	10.15	7.92
	AHSG	P29700	Alpha-2-HS-glycoprotein (Fragment)	8.48	8.98
	FLNA	A0A287AW81	Filamin A	3.56	4.37
	LRRC15	I3LCK1	Leucine rich repeat containing 15	3.56	-4.31
	GSN	P20305	Gelsolin (Fragment)	3.13	2.25
	A2M	A0A5G2Q8I9	Alpha-2-macroglobulin	3.25	4.08
	LOC100125542	A0A8W4FC14	Ig-like domain-containing protein	2.83	4.71
	TNNT3	Q75NG9	Troponin T, fast skeletal muscle	2.46	2.64
	VTN	P48819	Vitronectin	2.20	2.07
	ANXA5	F2Z5C1	Annexin	1.73	2.96
	CFD	P51779	Complement factor D	1.72	1.94
**Gastric**	**Gene**	**Uniprot ID**	**Protein name**	**log10 q-value**	**Effect Size**
	BLMH	F1RN71	Bleomycin hydrolase	12.07	1.92
	TGM1	K7GLF7	Protein-glutamine gamma-glutamyltransferase K	11.84	1.97
	C9H11ORF54	F1STK8	Chromosome 9 C11orf54 homolog	9.29	2.72
	AMY2	P00690	Pancreatic alpha-amylase	9.29	1.98
	ORM1	A0A5G2R0I4	Lipocalin/cytosolic fatty-acid binding domain-containing protein	8.70	6.67
	NAGK	A0A287B759	N-acetyl-D-glucosamine kinase	8.15	6.38
	LMNA	Q3ZD69	Prelamin-A/C	8.00	0.85
	F1SGI5	F1SGI5	IF rod domain-containing protein	7.04	2.87
	ALDH3A1	F1SDC4	Aldehyde dehydrogenase	7.04	2.08
	PSMB2	A0A5G2R6J1	Proteasome subunit beta	7.02	2.05
	GSDMA	F1RXA6	Gasdermin A	7.02	2.15
	DSG1	Q3BDI7	Desmoglein-1	7.02	2.07
	MYO5B	A0A5G2R6M1	Myosin VB	6.96	-2.86
	POF1B	F1S1P8	POF1B actin binding protein	6.89	1.28
	TNNT3	Q75NG9	Troponin T, fast skeletal muscle	6.85	2.40
	LGALSL	I3L8J2	Galectin	6.80	2.69
	RPN1	Q9GMB0	Dolichyl-diphosphooligosaccharide--protein glycosyltransferase subı	6.13	5.55
	KRT79	I3LLY8	Keratin 79	6.62	2.71
	TMEM131	A0A287BA74	Transmembrane protein 131	6.43	-2.86
	PANK4	I3LPY8	4'-phosphopantetheine phosphatase	6.20	-2.72
	ARG1	Q95JC8	Arginase-1	5.90	1.27
	JUP	Q8WNW3	Junction plakoglobin	5.59	0.79
	POFUT2	F1SG35	GDP-fucose protein O-fucosyltransferase 2	5.53	1.68
	LOC100125542	A0A8W4FC14	Ig-like domain-containing protein	5.25	3.11
	PSMA5	F2Z5K2	Proteasome subunit alpha type	5.22	1.20
	S100A16	F2Z5M4	Protein S100	5.21	1.58
	PLEKHO1	F1SS76	Pleckstrin homology domain containing O1	5.10	-3.41
	ENO1	A0A287B6S5	phosphopyruvate hydratase	5.09	1.84
	ACOXL	A0A287A1A8	Acyl-coenzyme A oxidase	5.09	-2.51
	ANXA2	P19620	Annexin A2	5.06	0.91
	SDR9C7	F1SL80	Short chain dehydrogenase/reductase family 9C member 7	5.06	1.18

DSC1	F1SAM0	Desmocollin 1	4.81	1.04
A0A5G2QLP2	A0A5G2QLP2	Secreted protein	4.79	-1.64
RAB10	F2Z5F2	RAB10, member RAS onco family	4.74	1.33
TMED10	A0A286ZV95	Transmembrane emp24 domain-containing protein 10	4.72	1.01
IQGAP3	A0A481A732	IQ motif containing GTPase activating protein 3	4.70	-2.62
HARS1	A0A8W4FL17	Histidine--tRNA ligase, cytoplasmic	4.69	1.11
ANXA1	P19619	Annexin A1	4.31	1.89
APEH	P19205	Acylamino-acid-releasing enzyme	4.23	1.53
ARHGEF40	I3LFZ9	Rho guanine nucleotide exchange factor 40	4.15	-1.54
WASF2	A0A5K1VSC0	Wiskott-Aldrich syndrome protein family member	4.12	1.32
EEF2	I3LII3	Eukaryotic translation elongation factor 2	4.11	1.24
EIF5A2	A0A287B4A3	Eukaryotic translation initiation factor 5A	3.97	1.68
C1ORF68	A0A8W4FGN1	Chromosome 1 open reading frame 68	3.91	-2.13
EIF2B5	I3LEF1	eIF-2B GDP-GTP exchange factor subunit epsilon	3.77	1.05
IQSEC1	A0A287BK53	IQ motif and Sec7 domain ArfGEF 1	3.77	-2.75
PLEC	A0A287A8C7	Plectin	3.75	1.14
B3GAT1	A0A286ZTI9	Galactosylgalactosylxylosylprotein 3-beta-glucuronosyltransferase	3.75	1.44
ALB	P08835	Albumin	3.66	2.25
NME1	A0A287AS29	Nucleoside diphosphate kinase	3.60	1.54
PSMA1	F2Z5L7	Proteasome subunit alpha type	3.59	1.22
BAIAP3	F1RFZ7	BAI1 associated protein 3	3.56	-1.17
AKR1B1	P80276;P82125	Aldo-keto reductase family 1 member B1	3.56	-1.35
PSMA7	A0A287AT18	Proteasome subunit alpha type	3.55	1.49
HSPA5	P34935	Endoplasmic reticulum chaperone BiP (Fragment)	3.39	1.34
NPM1	I3LUP6	Nucleophosmin	3.39	1.15
I3LHI7	I3LHI7	Peptidase S1 domain-containing protein	3.39	-0.96
RO60	A0A287B6J4	Ro60, Y RNA binding protein	3.35	1.11
SULT2B1	I3LRP8	Sulfotransferase	3.35	0.94
HSPA1B	Q6S4N2	Heat shock 70 kDa protein 1B	3.34	1.23
OBSCN	A0A8W4FKS7	Obscurin, cytoskeletal calmodulin and titin-interacting RhoGEF	3.26	1.20
RSKR	A0A286ZRK4	Ribosomal protein S6 kinase related	3.26	-1.06
CTSA	A0A480LAY6	Carboxypeptidase	3.23	1.44
PKP1	I3LGN8	Plakophilin 1	3.22	0.84
JHY	F1S9Q5	Junctional cadherin complex regulator	3.16	1.71
EDC4	F1S2J4	Enhancer of mRNA-decapping protein 4	3.13	-1.58
HAL	F1SQR7	Histidine ammonia-lyase	3.09	1.10
CTNNA1	A0A5G2R7Z4	Catenin alpha 1	2.99	-1.58
ASAH1	F1SES5	N-acylsphingosine amidohydrolase 1	2.85	1.18
CREBRF	F1SJY5	CREB3 regulatory factor	2.85	1.05
EIF6	I3L8I5	Eukaryotic translation initiation factor 6	2.83	0.89
RNF212	A0A5G2R093	RING-type domain-containing protein	2.78	-0.93
TOMT	F1SUX2	Transmembrane O-methyltransferase	2.69	-1.11
HAUS5	F1RM57	HAUS augmin like complex subunit 5	2.64	-0.88
IDH2	P33198	Isocitrate dehydrogenase [NADP], mitochondrial (Fragment)	2.63	1.04
GDI2	Q6Q7J2	Rab GDP dissociation inhibitor beta	2.62	0.77
CAPN1	P35750	Calpain-1 catalytic subunit	2.60	1.22
SMC4	A0A480E9Z3	Structural maintenance of chromosomes protein	2.59	-0.89
LOC100624590	A0A287A1E6;F1RX1	RNA helicase	2.56	0.92
A0A287AQB5	A0A287AQB5	Peptidase A1 domain-containing protein	2.54	1.14
VCP	P03974	Transitional endoplasmic reticulum ATPase	2.54	1.02
TERT	F1S027	Telomerase reverse transcriptase	2.49	-1.54
PADI1	K7GNB4	Protein-arginine deiminase	2.48	-0.79
AHSG	P29700	Alpha-2-HS-glycoprotein (Fragment)	2.46	1.21
ANXA5	F2Z5C1	Annexin	2.38	1.36
PSMB4	Q29384	Proteasome subunit beta type-4 (Fragment)	2.38	-0.76
PLA2G12A	F1S132	Phospholipase A2 group XIIA	2.28	1.23
SET	A0A5G2QKJ5	Protein SET	2.27	0.67
K7GLT8	K7GLT8	ATP synthase subunit beta	2.25	1.30
A0A8W4FQ74	A0A8W4FQ74	Reverse transcriptase domain-containing protein	2.22	1.15
PPL	F1RK90	Periplakin	2.20	-0.79
TF	P09571	Serotransferrin	2.16	1.04
ACTBL2	A0A287A4R1	Actin beta like 2	2.10	1.12
DDAH2	A0A287A5S7	dimethylargininase	2.07	0.65
HNRNPU	A0A286ZP67	Heterogeneous nuclear ribonucleoprotein U	2.05	0.96
PKM	A0A286ZI36	Pyruvate kinase	2.00	0.65

SMTN	F1RPD7	Smoothelin	1.96	-1.30
ACTR3	A0A286ZIH7	Actin related protein 3	1.94	0.75
TOP1MT	F1SEV4;I3LHM3	DNA topoisomerase I	1.88	-0.91
RPS15A	A0A287ADE6	40S ribosomal protein S15a	1.87	-1.10
HBA	P01965	Hemoglobin subunit alpha	1.80	1.46
MTFR2	F1S3R9	Mitochondrial fission regulator	1.79	1.02
CTSD	P00795	Cathepsin D	1.79	0.88
CAT	O62839	Catalase	1.71	0.81
KRT10	I3LDS3	Keratin 10	1.66	1.17
YBX3	I3LQF8	Y-box binding protein 3	1.52	0.65
VCL	P26234	Vinculin	3.30	0.58
PSMA3	F1SSL6	Proteasome subunit alpha type	3.12	0.52
IDE	A0A287BBS8	Insulin degrading enzyme	3.02	0.46
TPI1	Q29371	Triosephosphate isomerase	2.77	0.44
FLNA	A0A287AW81	Filamin A	2.49	0.43
MYH9	A0A8W4FCU2	Myosin heavy chain 9	2.02	0.46
CANX	A0A286ZSW6	Calnexin	1.76	0.59
PDCD6	A0A287AKU6	Programmed cell death 6	1.66	0.63
ANXA7	I3LEY2	Annexin	1.49	0.41
KRT77	A0A287BEI1	Keratin 77	1.46	0.50

Shows all significantly differentially expressed proteins identified per group. Columns in order as follows: Gene names from uniprot, Uniprot ID, Protein name, negative log10 q-value (FDR corrected p-value) as well as fold change.
